# Alternative Oxidase Gene Family in *Hypericum perforatum* L.: Characterization and Expression at the Post-germinative Phase

**DOI:** 10.3389/fpls.2016.01043

**Published:** 2016-08-11

**Authors:** Isabel Velada, Hélia G. Cardoso, Carla Ragonezi, Amaia Nogales, Alexandre Ferreira, Vera Valadas, Birgit Arnholdt-Schmitt

**Affiliations:** ^1^ICAAM - Instituto de Ciências Agrárias e Ambientais Mediterrânicas, Laboratório de Biologia Molecular, Universidade de ÉvoraPólo da Mitra, Évora, Portugal; ^2^Linking Landscape, Environment, Agriculture and Food, Instituto Superior de Agronomia-Universidade de LisboaLisboa, Portugal; ^3^EU Marie Curie Chair, ICAAM - Instituto de Ciências Agrárias e Ambientais Mediterrânicas, Universidade de ÉvoraPólo da Mitra, Évora, Portugal

**Keywords:** cyanide-resistant pathway, St. John's Wort, plant development, gene expression, transposable elements, miRNAs, alternative oxidase, post-germination

## Abstract

Alternative oxidase (*AOX*) protein is located in the inner mitochondrial membrane and is encoded in the nuclear genome being involved in plant response upon a diversity of environmental stresses and also in normal plant growth and development. Here we report the characterization of the *AOX* gene family of *Hypericum perforatum* L. Two *AOX* genes were identified, both with a structure of four exons (*HpAOX1*, acc. KU674355 and *HpAOX2*, acc. KU674356). High variability was found at the N-terminal region of the protein coincident with the high variability identified at the mitochondrial transit peptide. *In silico* analysis of regulatory elements located at intronic regions identified putative sequences coding for miRNA precursors and trace elements of a transposon. Simple sequence repeats were also identified. Additionally, the mRNA levels for the *HpAOX1* and *HpAOX2*, along with the ones for the *HpGAPA* (glyceraldehyde-3-phosphate dehydrogenase A subunit) and the *HpCAT1* (catalase 1), were evaluated during the post-germinative development. Gene expression analysis was performed by RT-qPCR with accurate data normalization, pointing out *HpHYP1* (chamba phenolic oxidative coupling protein 1) and *HpH2A* (histone 2A) as the most suitable reference genes (RGs) according to GeNorm algorithm. The *HpAOX2* transcript demonstrated larger stability during the process with a slight down-regulation in its expression. Contrarily, *HpAOX1* and *HpGAPA* (the corresponding protein is homolog to the chloroplast isoform involved in the photosynthetic carbon assimilation in other plant species) transcripts showed a marked increase, with a similar expression pattern between them, during the post-germinative development. On the other hand, the *HpCAT1* (the corresponding protein is homolog to the major H_2_O_2_-scavenging enzyme in other plant species) transcripts showed an opposite behavior with a down-regulation during the process. In summary, our findings, although preliminary, highlight the importance to investigate in more detail the participation of *AOX* genes during the post-germinative development in *H. perforatum*, in order to explore their functional role in optimizing photosynthesis and in the control of reactive oxygen species (ROS) levels during the process.

## Introduction

*Hypericum perforatum* L., (St. John's Wort) is a wide spread species found throughout all temperate regions of north and south hemispheres (Robson, 1977). Natural populations are usually observed in abandoned fields, along roadsides or in overgrazed rangelands (Maron et al., 2007). Its high interest for the pharmaceutical industry, due to its medicinal properties attributed to the presence of metabolites with an antidepressive, anticancer, and antiviral action (Zanoli, 2004; Gartner et al., 2005; Kubin et al., 2005), leads to a gradual re-expanding of field cultivation in Western Europe since the nineties.

Nevertheless, breeding of medicinal plants was neglected for a long time and has been initiated only about 20 years ago with special efforts in North-Europe. Homogeneous plantations are expected to provide farmers not only with higher yield stability, but also with increased homogeneity in product quality which in terms of medicinal plants would correspond to the composition of effective plant extracts. In this view, adequate technologies are being requested, especially the ones related with the control of seed germination, seedling development, and growth. Thus, fundamental and applied research can help to identify efficient strategies to reach this goal.

Marker assisted selection (MAS) is a process commonly used in plant breeding programs by which the selection of desirable phenotypic characteristics with agronomic interest, known as agronomic traits, is achieved indirectly by using DNA markers, closely linked to underlying gene(s), or developed from a specific gene(s). The last ones, named functional markers (FM), can be used to find out allelic variation in the genes underlying a trait, contributing to increase efficiency and accuracy of plant breeding programs, reason why have been gaining increasing attention during the last years (Andersen and Lübberstedt, 2003; Neale and Savolainen, 2004; Arnholdt-Schmitt, 2005). The identification of candidate genes is recognized as the first step for FM development. These genes can be found out by high-through-put differential gene expression analyses or by hypothesis-driven research approaches, which actually represent the most promising strategies in molecular plant breeding (Arnholdt-Schmitt, 2005; Collins et al., 2008). Alternative oxidase (*AOX*) was previously pointed out as a potential source for FM development related with plant plasticity under environmental stress, which means genotypes able to better succeed across variable conditions (Arnholdt-Schmitt et al., 2006; Clifton et al., 2006; Cardoso and Arnholdt-Schmitt, 2013) and have been also related to plant developmental processes (Campos et al., 2009, 2016; Santos Macedo et al., 2009).

AOX is a mitochondrial membrane protein acting as a terminal oxidase in the alternative (cyanide-resistant) respiratory pathway, where it reduces oxygen to water (Umbach et al., 2002). AOX allows continued turnover of carbon skeletons through glycolysis and the tricarboxylic acid (TCA) cycle when the cytochrome pathway is saturated, functioning as an overflow enzyme in the electron transport chain (ETC). However, even when the cytochrome pathway is not saturated, AOX may be activated in order to maintain balanced oxidation/reduction reactions and a balanced carbon metabolism (Rhoads et al., 1998 and references therein). AOX controls the formation of mitochondrial reactive oxygen species (ROS) and prevents specific components of the respiratory chain from over-reduction, thereby relieving oxygen species (OS) originated from environmental stresses (Popov et al., 1997; Amirsadeghi et al., 2007). Many reports show the involvement of AOX in plant response upon a diversity of biotic and abiotic stresses establishing the link between AOX and its role on the control of cellular ROS levels (see review Vanlerberghe, 2013). Other antioxidative enzymes are also implicated in the control of cellular ROS levels. Catalase (CAT), the major H_2_O_2_-scavenging enzyme in all aerobic organisms (Mhamdi et al., 2010), performs the rapid removal of H_2_O_2_ from the cell by oxidation of H_2_O_2_ to H_2_O and O_2_ (Møller et al., 2007). Nevertheless, ROS are not only involved in plant response upon stress conditions, they have also an active play in normal physiological processes such as in seed germination and in dormancy alleviation (Kwak et al., 2006; Bailly et al., 2008; Oracz et al., 2009). The ability of seeds to germinate might be related to their capacity to regulate ROS levels produced during mitochondrial oxygen consumption, and to neutralize the pro-oxidant activities of allelochemicals present in the medium (Pergo and Ishii-Iwamoto, 2011). It is expected that AOX, like other antioxidative enzymes, would be involved in the process of seed germination and seedling development by the control of ROS produced during the germination process, and besides also in the promotion of cellular homeostasis under the large metabolic changes that take place during germination and post-germination development. The process of seed germination starts when dry seeds get in touch with water under favorable conditions (imbibition), and it ends when radicle penetrates seed covering layers and is observable, followed by seedling establishment (Weitbrecht et al., 2011).

The step forward corresponds to seedling development (growth and differentiation). AOX has been implicated in the regulation of the mechanim of cell-reprogramming by improving metabolic transitions associated to the flexible carbon balance (Arnholdt-Schmitt et al., 2006; Rasmusson et al., 2009). Several authors have shown that regulation of soybean *AOX* genes depend on the post-germinative development of soybean cotyledons (Finnegan et al., 1997; McCabe et al., 1998). In some plants, cyanide-insensitive respiration is required for germination (reviewed by Botha et al., 1992). For example, in cocklebur, the alternative pathway was activated after imbibition of seeds (Esashi et al., 1981; Saisho et al., 2001).

Considering this previous knowledge, this study aimed to isolate and characterize the *H. perforatum AOX* gene family to then be able to explore, by *in silico* analysis, the existence of regulatory elements located at intronic regions. Also, to investigate at the mRNA level the expression of the *AOX* gene family members during the post-germinative development along with two other proteins know to be involved, in other plant species, in different pathways: CAT1 involved in the control of ROS levels and chloroplast glyceraldehyde-3-phosphate dehydrogenase A subunit (GAPA) involved in the photosynthetic carbon assimilation.

## Materials and methods

### *HpAOX* gene member's identification and isolation

#### Isolation of complete *HpAOX* genes

Complete *AOX* gene isolation was performed in several steps. First, partial gene isolation was performed using degenerated P1 and P2 primers following the protocol previously described by Saisho et al. (1997). Genomic DNA (gDNA) used as template in the PCR was extracted from 1 month old seedlings (bulked of eight seedlings previously established under *in vitro* conditions) using the DNeasy Plant Mini Kit (Qiagen, Hilden, Germany) according to the manufacturer's protocol. PCR was conducted with the Ready-To-Go PCR Beads (GE Healthcare, Little Chalfont, England) using 10 ng of gDNA and 0.2 μM of each primer. PCR was carried out for 35 cycles in the 2770 thermocycler (Applied Biosystems, Foster City, CA, USA).

In a second step, the 5′ and 3′ ends of the isolated *HpAOX* gene fragments were determined by 5′ and 3′ RACE-PCRs. For this, total RNA was extracted, from the same material used for gDNA extraction, using the RNeasy Plant Mini Kit (Qiagen, Hilden, Germany) with on-column digestion of DNA applying the RNase-Free DNase Set (Qiagen, Hilden, Germany), according to manufacturer's protocol.

For 3′ end isolation, a cDNA single strand was produced by RevertAid HMinus First Strand cDNA Synthesis kit (Fermentas, Ontario, Canada) according to manufacturer's instructions with oligo d(T) primer (Roche, Mannheim, Germany) and 5 μg of total RNA. 3′RACE-PCR was conducted using 1 μl of cDNA single strand as template with the reverse primer *VIAL 9* (Roche, Mannheim, Germany) and a gene-specific forward primer (see sequence on Table S1).

For 5′ end isolation, 1 μg of total RNA was used to synthesize the first-strand cDNA using the SMARTer™ RACE cDNA Amplification kit (Clontech Laboratories, Inc., Mountain View, CA, USA) according to manufacturer's instructions. 5′RACE-PCR was carried out using 0.2 μM of the reverse gene specific primer (Table S1) following protocol provided with the kit.

Finally, for the complete gene isolation, gDNA and total RNA were isolated from a single *in vitro* growing plantlet following the procedures described above. One gene-specific primer set was designed for each *HpAOX* gene (see Table S1) based on the previously isolated 5′ and 3′-UTR sequences. Ten nanograms of gDNA or 1 μl of oligo d(T) first strand cDNA were used as template with 0.2 μM of each specific primer. PCRs were performed using Phusion High-Fidelity DNA Polymerase (Finnzymes, Espoo, Finland) according to the manufacturer's protocol (see annealing temperatures at Table S1).

#### Cloning and sequence analysis

PCR fragments were separately cloned into a pGem®-T Easy vector (Promega, Madison, USA) and used to transform *E. coli* JM109 (Promega Madison, WI, USA) competent cells. Plasmid DNA was further extracted from putative recombinant clones by alkaline lysis protocol (Bimboim and Doly, 1979) and sense and antisense strands were sequenced (Macrogen company: www.macrogen.com) using primers T7 and SP6 (Promega, Madison, USA).

Sequence homology was searched in the NCBI databases (National Center for Biotechnology Information, Bethesda, MD) using BLAST algorithm (Karlin and Altschul, 1993; http://www.ncbi.nlm.nih.gov/; BLASTn and BLASTp).

CLC Main Workbench 7.5.1 software (ClCbio, Aarhus N, Denmark) was used to edit *HpAOX* sequences (cDNA, gDNA and putative translated peptide). Intron location was made using the software Spidey (http://www.ncbi.nlm.nih.gov/IEB/Research/Ostell/Spidey/). Gene draw was performed in FancyGene 1.4 (Rambaldi and Ciccarelli, 2009), freely available at http://bio.ieo.eu/fancygene/.

Sequences were aligned in MUSCLE (http://www.ebi.ac.uk/Tools/msa/muscle/) following the standard parameters. Phylogenetic reconstruction was performed in MEGA software (Tamura et al., 2007) by Neighbor-Joining (NJ) and the inferred tree was tested by bootstrap analysis using 1000 replicates. Graphical view was edited in the Fig Tree v14.0 software (http://tree.bio.ed.ac.uk/software/figtree/). The free available TargetP software (Emanuelsson et al., 2000) was used to predict the protein subcellular localization and the position of the cleavage sites of mitochondrial targeting signals (http://www.cbs.dtu.dk/services/TargetP/).

#### *In silico* identification of regulatory elements located at the *HpAOX* intronic regions

For the identification of putative miRNA precursor sequences located at the *HpAOX* introns, the publicly available software miR-*abela* (http://www.mirz.unibas.ch/cgi/pred_miRNA_genes.cgi) was used. MiPred software was used (http://server.malab.cn/MiPred/; Jiang et al., 2007) to validate potential pre-miRNAs and the web-based software Mfold, (available at http://mfold.rit.albany.edu/?q=mfold/RNA-Folding-Form; Zuker, 2003) applied in the prediction of the secondary structure of pre-miRNA. To screen potential miRNAs candidates, the previous validated pre-miRNA sequences were analyzed with the software miRBase (http://www.mirbase.org/search.shtml). BLASTx from NCBI database (http://www.ncbi.nlm.nih.gov/BLAST/) was used to find the potential target genes (Mathews et al., 1999; Zuker, 2003).

For identification of transposable elements (TE) putatively located at the intronic regions of *HpAOX*, the CENSOR software tool from the Genetic Information Research Institute—GIRI (http://www.girinst.org/censor/index.php) was used (Kohany et al., 2006). For identification of simple sequence repeats (SSRs) the RepeatMasker platform was used (http://www.repeatmasker.org/; Smit et al., 2013-2015).

### *HpAOX* transcript quantification

#### Plant material and experimental conditions

In order to evaluate the role of *AOX* genes in *H. perforatum* post-germinative development an experiment was conducted. Seeds were collected from the achenes of a mother plant growing in the field (Viana do Alentejo, Portugal, 38°21′37″N, 7°59′13″W). No specific licenses were needed for achene harvesting since *H. perforatum* is not a protected species. Seeds were inoculated *in vitro* after their disinfection (see details in Ferreira et al., 2009) and maintained during a period of 14 days under 16 h photoperiod, constant day/night temperature of 25°C, and 80 μmol m^−2^s^−1^ of light intensity (Philips fluorescent lamps).

**Figure 1:**
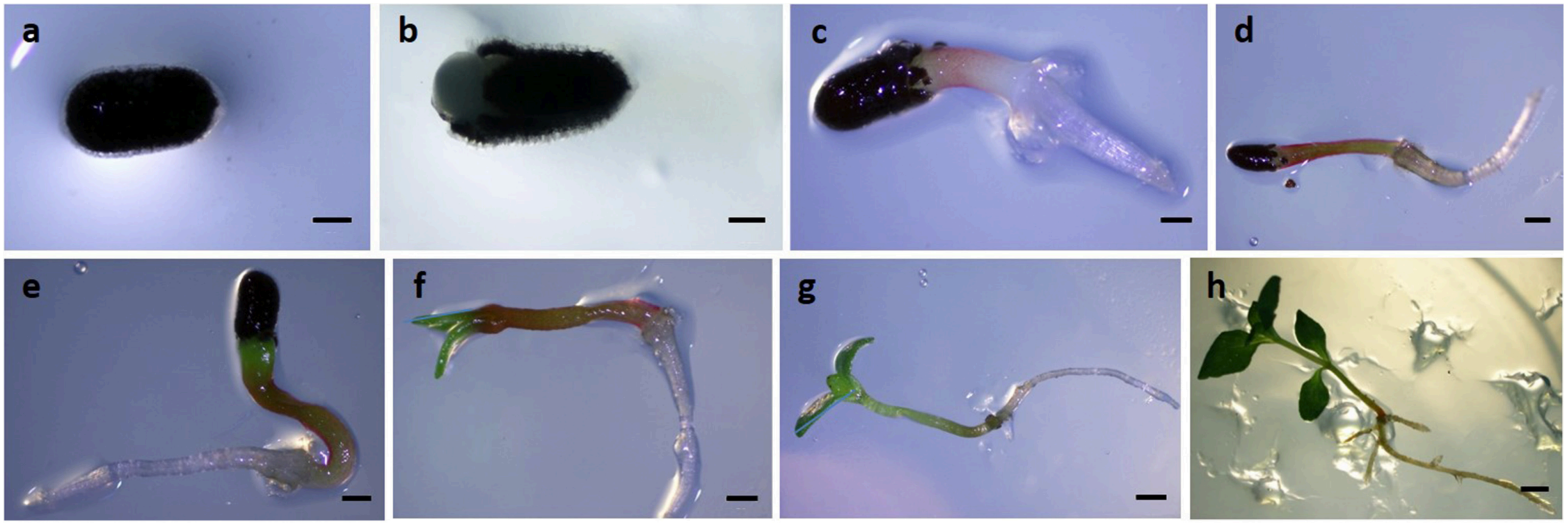
**Seed germination and post-germinative development of seedlings in ***Hypericum perforatum***. (A)** 0 days, **(B)** 2 days, **(C)** 4 days, **(D)** 6 days, **(E)** 8 days, **(F)** 10 days, **(G)** 12 days, **(H)** 14 days post-sowing. Bars: **(A–C)** = 0, 5 mm; **(D–G)** = 1 mm; **(H)** = 5 mm.

Samples were collected at different time points: 4, 6, 8, 10, 12, and 14 days after sowing (Figure 1). Samples collected between 4 and 8 days consisted in a bulked sample of ~50 explants. Samples collected between 10 and 14 days represented a set of ~10 seedlings for each time point.

#### RNA isolation and first-strand cDNA synthesis

Total RNA was extracted with the RNeasy Plant Mini Kit (Qiagen, Hilden, Germany), according to the manufacturer's instructions, and eluted in 30 μl volume of RNase-free water. Possible residual genomic DNA present in RNA samples was digested with the DNase I (RNase-Free DNase Set, Qiagen, Hilden, Germany), following the manufacturer's instructions. The RNA concentration was assessed with the NanoDrop-2000C spectrophotometer (Thermo Scientific, Wilmington, DE, USA), and the RNA integrity was checked, based on the presence of the two ribosomal subunits, by agarose gel electrophoresis in a Gene Flash Bio Imaging system (Syngene, Cambridge, UK). Maxima First Strand cDNA Synthesis Kit for RT-qPCR (Thermo Scientific, Wilmington, DE, USA) was used to synthesize complementary DNA (cDNA) from RNA samples (using 1 μg of total RNA), according to the manufacturer's instructions.

#### Quantitative real-time PCR

Real-time PCR was performed in the Applied Biosystems 7500 Real-Time PCR System (Applied Biosystems, Foster City, CA, USA). Real-time PCR reactions were carried out using 1X Maxima SYBR Green qPCR Master Mix, 300 nM of forward and reverse primers, and 1.25 ng of cDNA in a total volume of 18 μl. Primers for the 11 genes analyzed here were designed based on the *H. perforatum* sequences deposited in the NCBI with the Primer Express v3.0 (Applied Biosystems, Foster City, CA, USA), using the default parameters of the software (Table 1). All primer pairs were evaluated for their probability to form dimers and secondary structures using the proper tool of the software for the purpose. The following thermal profile was applied: 10 min at 95°C, and 40 cycles of 15 s at 95°C and 60 s at 60°C. Possible contaminations and primer dimers formation were discarded by using no-template controls (NTCs). A standard curve with four points was performed using the undiluted pool containing all cDNA samples and three 5-fold serial dilutions. All samples were run in duplicate. A melting curve analysis was performed to guarantee amplification of specific products. The values of quantification cycles (C_q_) were obtained for each sample with the Applied Biosystems 7500 software (Applied Biosystems, Foster City, CA, USA).

**Table 1:** **Primer sequences and other parameters for the genes used in this study**.

**Gene (acc. no.)**	**Primer Sequence (5′–3′)**	**AL (bp)**	**Tm (°C)**	***r*^2^/E (%)**
*Hp18SrRNA* (AF206934)	Fw: CGTCCCTGCCCTTTGTACAC	72	80.23	0.999/94.50
	Rv: CGAACACTTCACCGGACCAT			
*Hp26SrRNA* (DQ110887)	Fw: GCGTTCGAATTGTAGTCTGAAGAA	65	80.79	0.999/90.65
	Rv: CGGCACCCCCTTCCAA			
*HpGAPA* (EU301783)	Fw: GGTCGACTTCAGGTGCAGTGA	76	81.04	0.999/83.88
	Rv: CACCATGTCGTCTCCCATCA			
*HpGSA* (KJ624985)	Fw: GCAATAATCCTTGAACCTGTTGTG	78	78.35	0.992/94.30
	Rv: CCTGCGGAGAGCGTTGA			
*HpHYP1* (JF774163)	Fw: GGAGGAAGCAAGGGTAAGATTACA	81	77.18	0.997/93.97
	Rv: CCCGATCTTGACTTCTTCTTCATT			
*HpH2A* (EU034009)	Fw: CCGGTTGGGAGGGTTCA	63	79.64	0.995/95.01
	Rv: TGCACCGACCCTTCCATT			
*HpRBCL* (HM850066)	Fw: CGCGGTGGGCTTGATTT	71	76.86	0.999/98.95
	Rv: CGATCCCTCCATCGCATAAA			
*HpTUB* (KJ669725)	Fw: GGAGTACCCTGACAGAATGATGCT	80	77.89	0.990/93.48
	Rv: TTGTACGGCTCAACAACAGTATCC			
*HpAOX1* (EU330415)	Fw: TTGGACAATGGCAACATCGA	69	80.11	0.995/90.46
	Rv: GGGAGGTAGGCGCCAGTAGT			
*HpAOX2* (EU330413)	Fw: TCAACGCCTACTTTGTGATCTATCTC	80	78.47	0.998/95.03
	Rv: AATGGCCTCTTCTTCCAAATAGC			
*HpCAT1* (AY173073)	Fw: CGCTTCCTCAACAGATGGATTAG	71	79.10	0.996/96.45
	Rv: ACCCAGATGGCTCTGATTTCA			

*acc. no., NCBI accession number; AL, amplicon length; Tm, melting temperature for each amplicon; r^2^, correlation coefficient; E, PCR efficiency*.

#### Determination of reference gene expression stability using GeNorm algorithm

GeNorm algorithm was used to determine the expression stability of each candidate reference gene (RG). The input data for GeNorm algorithm were the relative quantities (RQ) calculated with the Cq value for each sample by the delta-Ct method using the formula RQ = E^ΔCq^, where E is the amplification efficiency calculated for each primer pair and ΔC_q_ = lowest C_q_ – sample C_q_ (Vandesompele et al., 2002). Amplification efficiency (E) was calculated using the formula *E* = 10^(−1∕slope)^ where the slope value was given by the Applied Biosystems (AB) software. GeNorm determines the pairwise variation (*V*; Vandesompele et al., 2002) and in the present study *V* values were determined for the candidate RGs using a cut-off value of 0.15, below which the inclusion of an additional RG is not required for normalization. In this way, GeNorm also determines the optimal number of genes required to calculate a reliable normalization factor.

#### Analysis of transcript expression

For expression levels normalization of the genes under study, C_q_ values were converted into RQ by the delta-Ct method, as described in previous section. The normalization factor was determined by the GeNorm algorithm and corresponds to the geometric mean between the relative quantities of the selected RGs, for each sample. For each gene of interest, the normalized value of gene expression is obtained by doing the ratio between the relative quantities and the corresponding normalization factor, for each sample. Graphics indicate the mean ± standard deviation of three biological replicates. The control group (4 dps) was set to 1 and the bars corresponding to the other time points show the fold-change with respect to 4 dps. The *t*-test method [IBM® SPSS® Statistics version 22.0 (SPSS Inc., USA)] was used to ascertain statistical significances (*p* ≤ 0.05 and *p* ≤ 0.01) between means.

## Results

### Characterization of the full-length sequences of *HpAOX* gene family

The use of P1/P2 primers allowed the isolation of amplicons with 444 bp length showing high homology with *AOX* genes from other plant species, which was expected considering the previous identification of *AOX* genes using this degenerated primer pair not only in *Arabidopsis thaliana* (Saisho et al., 1997) but also in several other plant species (Campos et al., 2009; Costa et al., 2009; Frederico et al., 2009; Santos Macedo et al., 2009) where it is included *H. perforatum* with three *AOX1* gene members and a single *AOX2* (Ferreira et al., 2009). However, the use of amplicon-specific primers located at 5′ and 3′ends allowed the isolation of the full-length cDNA sequences from only two *HpAOX* genes. After several attempts it was possible to confirm the existence of only two *AOX* gene members in *H. perforatum*, one belonging to the *AOX1*-subfamily and another to the *AOX2*-subfamily.

Gene size variability was identified at cDNA level in both genes. 5′ end showed variability among sequences of the same gene. In the case of *HpAOX1*, the length of 5′ end ranged between 26 and 116 bp (Figure S1), and in *HpAOX2* it ranged between 56 and 111 bp (Figure S2). Sequence size variability was also detected at 3′ end of the *HpAOX1* ranging between 159 and 227 bp (Figure S3). The larger sequences are presented in Figures S4, S5.

The *AOX1* gene at cDNA level isolated from *H. perforatum* (deposited at the NCBI with the acc. EU330415.2) has 1415 bp (Figure S4) and presents an open reading frame (ORF) of 1056 bp, which encodes a putative polypeptide with 351 amino acid residues. The *AOX2*-subfamily member, *HpAOX2* (acc. EU330413.2), presents 1311 bp and an ORF of 1017 bp encoding a putative polypeptide with 338 amino acid residues (Figure S5). The first ATG codon found in the beginning of the resultant ORF of *HpAOX1* represents the correct initiation of translation. In the *HpAOX2* two putative start codons were detected, one generating a larger peptide (with more 18 amino acid residues, see Figure S2). However, no similarity with other plant species was observed on that region which lead us to select a sequence encoding the shorter sequence. Figures S4, S5 indicate the cDNA sequences for both genes including the putative translated peptide and the conserved sites for intron positions. The difference in the overall length for the complete ORF sequences is mostly due to the size variability at exon 1 in the N-terminal region. Exon 1 has a size of 378 bp for *HpAOX1* and 339 bp for *HpAOX2*.

**Figure 2:**
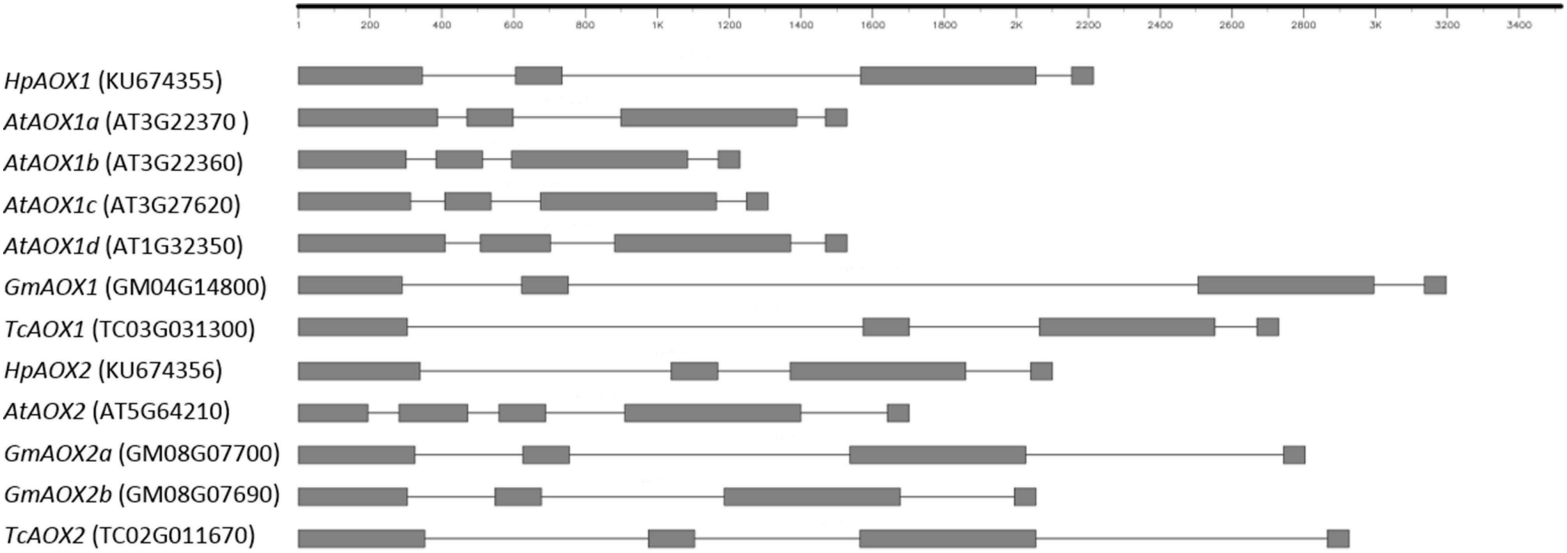
**Schematic representation of the structure identified in ***Hypericum perforatum AOX*** genes in comparison with genes from plant species that show different patterns of ***AOX***-subfamilies ramification: ***Arabidopsis thaliana*** characterized by ramification of ***AOX1***-subfamily, Glycine max by ramification of ***AOX2***-subfamily and ***Theobroma cacao*** with a single gene in both sub-families**. Sequences were collected from Plaza: http://bioinformatics.psb.ugent.be/plaza/; Gene draw was performed in FancyGene 1.4 (Rambaldi and Ciccarelli 2009), freely available at http://bio.ieo.eu/fancygene/.

Forward and reverse primers located at 5′ and 3′ gene ends, respectively, were used at genomic level and allowed the isolation of *HpAOX1* gene with 2825 bp and *HpAOX2* with 2213 bp. Gene structure, common to both *HpAOX* genes, is composed by four exons interrupted by three introns (Figure 2). The conservation of exons size is here confirmed for the three last exons (exon 2: 129, exon 3: 489, and exon 4: 57 bp). Contrarily to this conservation at the exons, high level of variability in intronic regions was observed. Intron 1, intron 2, and intron 3 of *HpAOX1* are 261, 831, and 99 bp long, respectively, while in *HpAOX2* they are 699, 202, and 181 bp long, respectively. This variability was observed within a gene and across gene members from the *AOX* subfamily/family at species level and across species (Figure 2).

**Figure 3:**
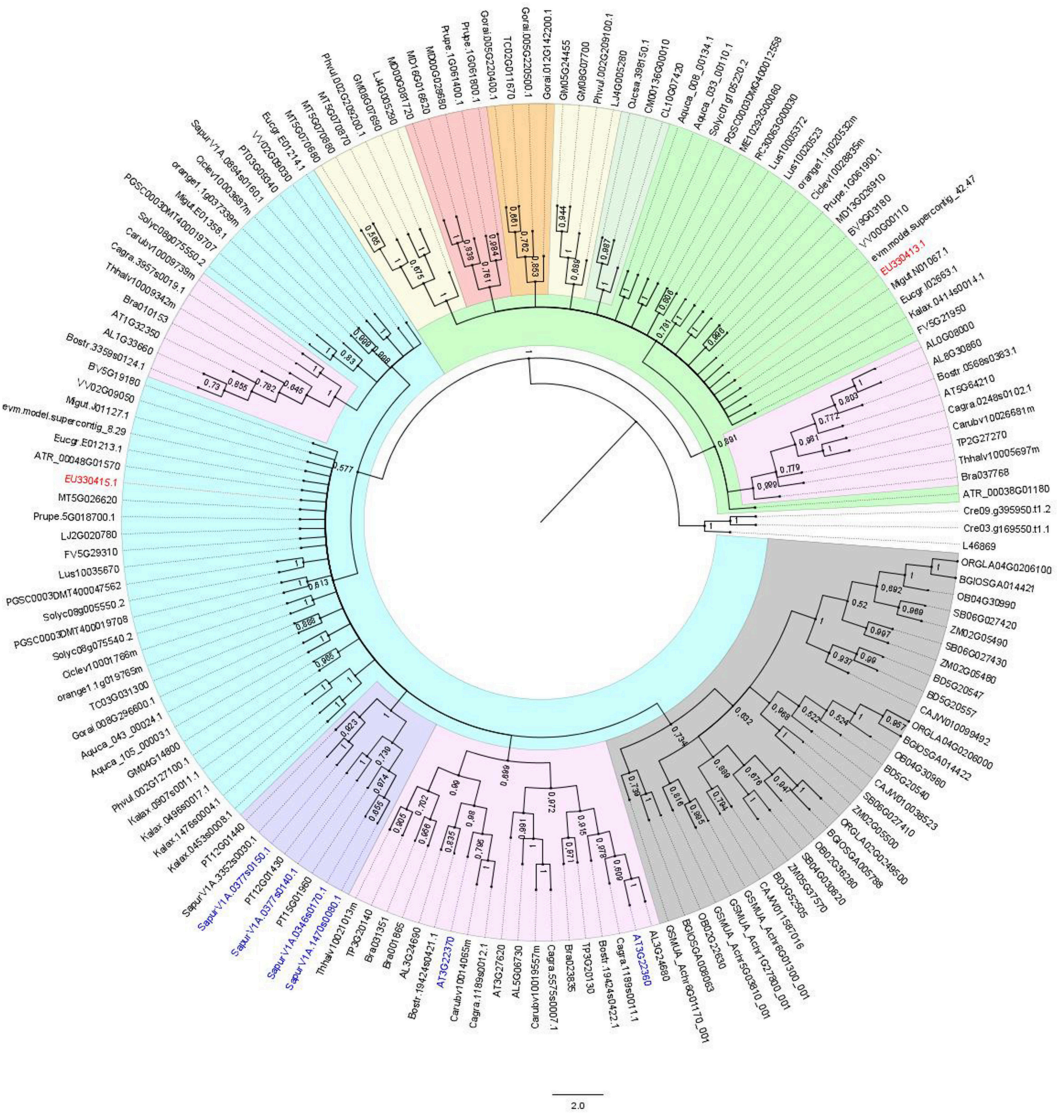
**Neighbor-Joining (NJ) tree showing the relationships among deduced AOX sequences from 45 plant species, including monocot and eudicot plant species**. Both AOX sequences of *Hypericum perforatum* were included (in red). 153 AOX sequences from higher plants were included (correspondence of accession numbers and the plant species is included in Tables S2, S3). The NJ tree was obtained using the complete peptide sequences. The alignments were bootstrapped with 1000 replicates by the NJ method using the MEGA 6 software. AOX sequence from *Neurospora crassa* and two sequences of *Chlamydomonas reinhardtii* were used as outgroup. The scale bar indicates the relative amount of change along branches. In blue the branch corresponding to the AOX1-subfamily and in green the branch corresponding to the AOX2-subfamily. In yellow the branch corresponding to the AOX1d.

The homologous identity score obtained with the deduced amino acid residue sequence revealed that both *Hp*AOX1 and *Hp*AOX2 share a great similarity with other AOX proteins, like AOX1a from *Arabidopsis thaliana* (acc. NP_188876.1, 70%) and AOX2 from *Vitis vinifera* (acc. NP_001268001.1, 71%), respectively. To determine the relationship between both *HpAOX* genes and *AOX* from other eudicot plant species a NJ tree was constructed using the 153 translated AOX sequences from both eudicot and monocot plant species (Figure 3). A clear separation between both *AOX*-subfamilies could be seen forming the two main clusters (in blue the *AOX1*-subfamily and in green the *AOX2*-subfamily). Each *HpAOX* member was grouped in each *AOX*-subfamily. Monocot *AOX1* members form a separated group within the cluster of the *AOX1*-subfamily. Gene members from species that belong to the same family also group in separated clusters within each subfamily. A syntheny plot to the *AOX* gene family (HOM03D001776) across whole genomes available at PLAZA 3.0 and Phytozome v11.0 showed that *AOX* genes occur mainly as a single copy gene in sense or reverse orientation. Some cases of tandem duplication events are also reported. In the NJ tree it is possible to see that translated peptides corresponding to those duplicated events are not always grouped together (examples are highlighted in blue). Sequences of *Salix purpurea* are tandem repeats in which two sequences group together (SapurV1A.1470s0080.1 and SapurV1A.0346s0170.1) and another not (SapurV1A.0377s0140.1.p and SapurV1A. 0377s0150.1.p). A second example are the sequences of *A. thaliana* (AT3G22360.1 and AT3G22370.1) which share more similarity with sequences of other plant species.

A multiple sequence alignment, constructed using complete AOX sequences from plant species including both *Hp*AOX deduced sequences (*Hp*AOX1 and *Hp*AOX2), allowed us to highlight similarities and differences in the protein sequences (Figure S6). The predicted cleavage site length of the mitochondrial transit peptide (mTP) from the start of the protein is highlighted in blue boxes for all sequences included in the alignment. A great variation on the predicted length of this region can be seen, going from 8 amino acids in AtAOX1b (acc. AT3G22360) to 72 amino acids in AtAOX2 (acc. AT5G64210). Both *Hp*AOX proteins were predicted to be located in the mitochondria (mit score of 76% for *Hp*AOX1 and 87% for *Hp*AOX2).

Helices α1 and α4 (in red in Figure S6) that make part of the hydrophobic region of the AOX molecular surface demonstrated high degree of variability when compared with helices α2, α3, α5, and α6 (in green in Figure S6) which form the four-helix bundle. The helix α4 is slightly more conserved than α1. In fact, low level of similarity can be seen at the N-terminal region, not only related with differences in size (shown by the presence of minus signs) due to exon 1 variability reported above, but also due to different amino acid composition (shown by the presence of amino acids colored in red). Increase in the similarity, at both levels (sequence composition and size conservation), starts near the conserved regulatory cystein residue I (CysI). All main features that characterize AOX proteins are equally conserved at both *Hp*AOX peptide sequences. There are examples, the four universally conserved glutamate residues (E) and the two universally conserved histidine (H) residues, and the amino acid residues that interact with the protein inhibitor located at helix α5. *In silico* analysis searching for regulatory elements lead us to the identification of simple sequence repeats (SSR) in intronic regions of *HpAOX1*: a 59 bp long TTTAT motif in intron 1 and a 32 bp long AT motif in intron 2, and of *HpAOX2*: a 44 bp long CGGAGG motif located at exon 1 (location of SSRs are presented in Figures S7, S8 of Supplementary material). CENSOR software detected a region of 119 bp in the *HpAOX2* sequence with similarity to the LTR region of a *Cassandra* MT-LTR retrotransposon from *Medicago truncatula*. RepeatMasker software also found a positive match of this fragment with a *Cassandra* LTR from *Zea mays*. A blast search in the NCBI databases using that previous identified sequence revealed a 72% homology to a *Pisum sativum Cassandra*-like LTR.

Additionally, a search for putative miRNA precursor sequences (pre-miRNAs) identified one putative pre-miRNA located at intron 1 and two pre-miRNAs at intron 2 of *HpAOX1* (Table 2). Potential candidates for miRNAs were identified with homology with miRNAs previously described in other plant species. The pre-miRNA at intron 1 revealed homology with the gma-miR5780c; the pre-miRNAs at intron 2 revealed homology with vvi-miR3637-5p and gra-miR7484n. These homologous miRNAs seem to be encoded by pre-miRNA sequences located also at intronic regions (see Figure S9). No target genes were identified for any of the predicted miRNAs.

**Table 2:**
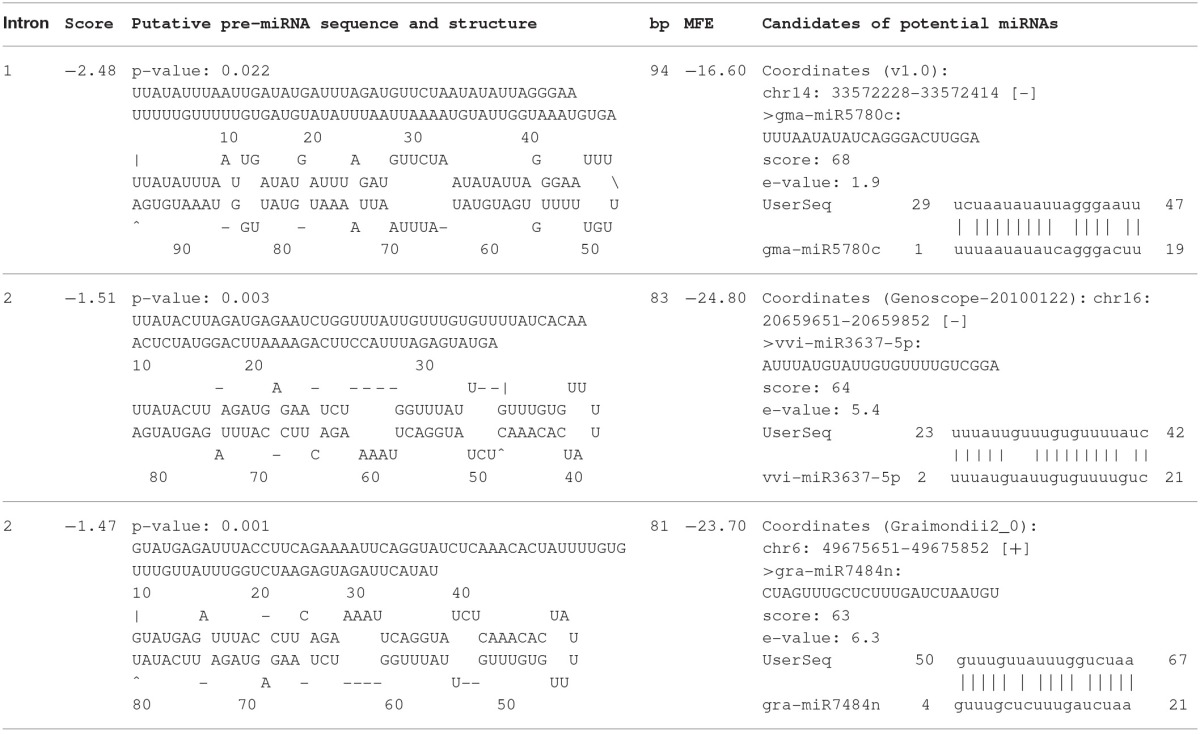
**Computational prediction of intronic miRNA precursors in ***AOX*** genes of ***Hypericum perforatum*** determined at miR-abela software (http://www.mirz.unibas.ch/cgi/pred_miRNA_genes.cgi) using as prediction threshold-10**.

*Score, given at miR-abela software to the identified pre-miRNA sequence; bp, length of the pre-miRNA sequence in bp; MFE, minimal free energy in kcal/mol. Prediction confidence to be a pseudo microRNA precursor was calculated at MiPred software (shuffle times: 1000) as 100% (http://server.malab.cn/MiPred/; Jiang et al., 2007). For screening the candidates of potential miRNAs the validated pre-miRNA were run in the software miRBase (http://www.mirbase.org/search.shtml)*.

### Selection of reference genes

The analysis of expression stability was performed with the following candidate reference genes: glyceraldehyde-3-phosphate dehydrogenase A subunit (*GAPA*), 18S (*18SrRNA*) and 26S (*26SrRNA*) ribosomal RNAs, beta-tubulin (*TUB*), ribulose-1,5-bisphosphate carboxylase/oxygenase large subunit (*RBCL*), glutamate-1-semialdehyde 2,1-aminomutase (GSA), chamba phenolic oxidative coupling protein (*HYP1*), and histone 2A (*H2A*). This analysis was performed to select the most suitable ones to be used to normalize the expression levels of target genes. GeNorm algorithm selected *HpHYP1* and *HpH2A* (*M* = 0.347) simultaneously as the most stable genes (Figure S10A) with no need to include a third RG for normalization (Figure S10B). *HYP1* is involved in plant defense and *H2A* participates in nucleosome assembly.

The amplification specificity for each gene was confirmed by the melting curve analysis, observing amplification of the expected specific product and no formation of primer dimers (Figure S11). The slope and correlation coefficient (*r*^2^) values were given by the AB software. The *r*^2^ ranged from 0.990 to 0.999 and the slope values ranged between −3.780 (PCR efficiency = 83.9%) for *HpGAPA* and −3.347 (PCR efficiency = 98.9%) for *HpRBCL* (Table 1). The PCR efficiency for *HpGAPA* revealed to be low, nevertheless, and due to the fact the corresponding *r*^2^ was high (0.999), the gene was not discarded from the analysis. The PCR efficiency value for *HpGAPA* was taken into account in the formula for the RQ calculation.

### Analysis of transcript expression

The transcript expression profiles of *HpAOX1, HpAOX2, HpCAT1*, and also *HpGAPA* were analyzed in the present work. The amplification specificity of the genes was verified by the melting curve analysis (Figure S11). The geometric mean of the 2 top-ranked RGs (*HpHYP1* and *HpH2A*), given by the GeNorm algorithm, was used in gene expression data normalization, as described above in the “Selection of reference genes” section.

**Figure 4:**
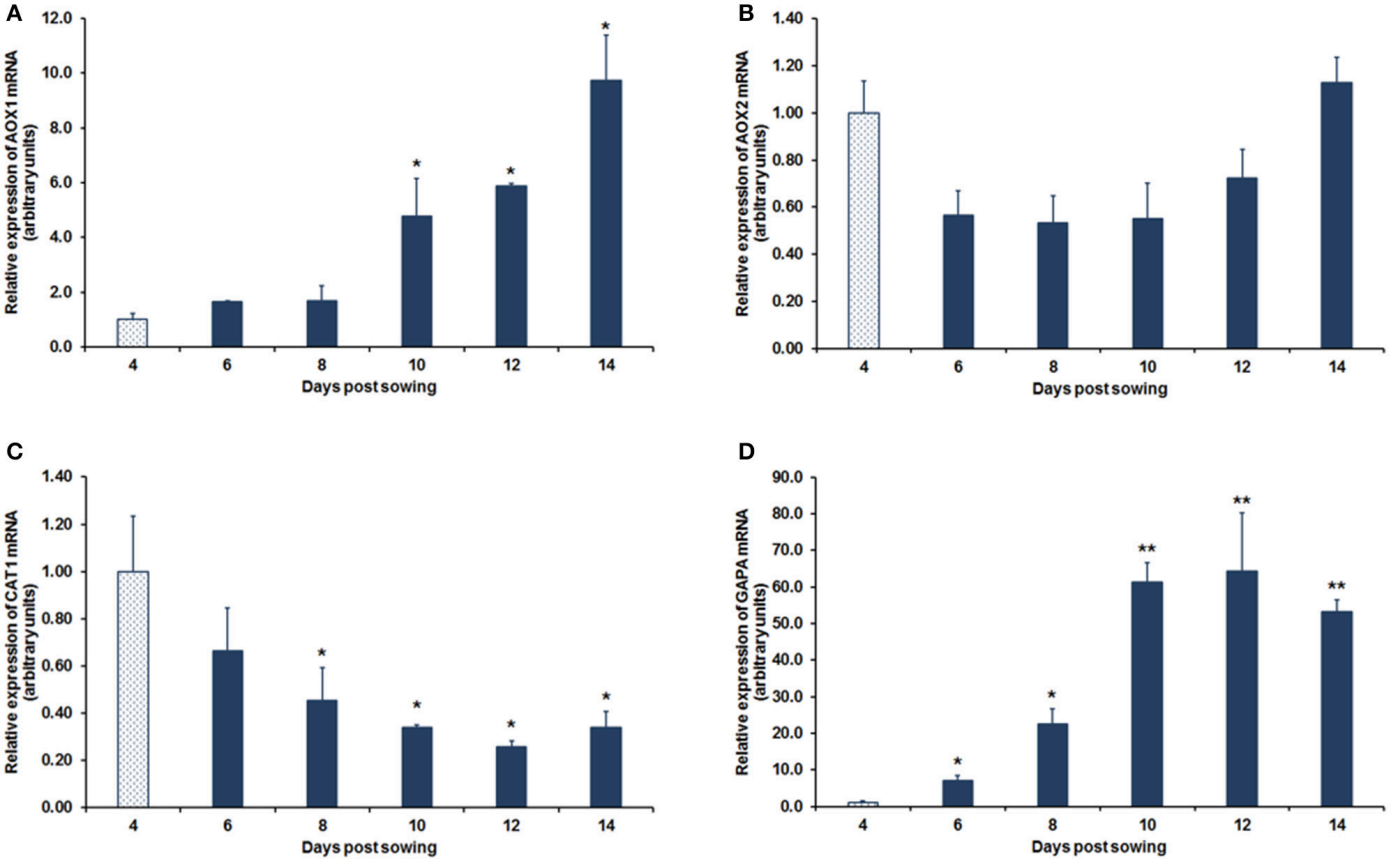
**Relative mRNA expression during post-germinative development of ***H. perforatum*** seedlings**. Transcript expression of *HpAOX1* **(A)**, *HpAOX2* **(B)**, *HpCAT1* **(C)**, and *HpGAPA* **(D)**. *HpHYP1* and *HpH2A* were used as reference genes in data normalization. The relative expression values are depicted as the mean ± standard deviation of three biological replicates for each time point. The bars represent the fold-change related to the time point 4 days post-sowing, which was set to 1. Statistical significances (^*^p ≤ 0.05 and ^**^p ≤ 0.01) between the two means were determined by the *t*-test using IBM® SPSS® Statistics version 22.0 (SPSS Inc., USA).

The mRNA of *HpAOX1* was maintained constant from day 4 after seed *in vitro* sowing (dps, days post-sowing) up to day 8, nevertheless, after this time point, an accumulation of the transcript was observed up to 14 dps with an increase of approximately 10-fold (*p* ≤ 0.05; Figure 4A). The mRNA expression of *HpAOX2* did not differ statistically over time in the post-germination process, even if a slight tendency to decrease (1.8-fold-change) from day 4 to 6 after seeds sowing was observed. This expression was maintained up to day 10 post-sowing and a subsequent complete recovery was observed up to day 14, demonstrating high stability of this gene during the developmental process (Figure 4B). The expression of *HpCAT1* transcript showed a gradual down-regulation over the 12 dps of about 3.8-fold (*p* ≤ 0.05) which was maintained up to 14 dps (Figure 4C). The *HpGAPA* transcript increased drastically about 64-fold (*p* ≤ 0.01) from day 4 up to day 12, maintaining the expression at elevated levels up to day 14 (Figure 4D).

## Discussion

### *HpAOX* genes sequence analysis

Mitochondrial AOX proteins are encoded by a small nuclear gene family, with a maximum of six gene members (Cardoso et al., 2015) distributed in 2 subfamilies, *AOX1* and *AOX2* (Considine et al., 2002; Borecky et al., 2006). In higher plants *AOX1* is found in all studied monocot and eudicot plant species while *AOX2* is only present in eudicots (Considine et al., 2002). The number of *AOX* gene members and its pattern of expansion is highly variable across eudicot plant species (Cardoso et al., 2015). At genome level, *AOX* genes appear usually as single copy genes. Nevertheless, 17 cases of duplication events can also be seen in different plant species. The majority of these duplicated copies are adjacent to the original (tandem repeats, e.g., SapurV1A.0377s0140.1.p and SapurV1A. 0377s0150.1.p). Three exceptions appear with the duplicated sequence located somewhere along the chromosome (e.g., SapurV1A.1470s0080.1 and SapurV1A.0346s0170.1). Tandem duplication events have been described as one of the major mechanisms that creates new genes, particularly in cases where genes are clustered into a gene family (Fan et al., 2008). In *H. perforatum* it was identified the complete sequence of two *AOX* genes, one belonging to the *AOX1*-subfamily and the other to the *AOX2*-subfamily. Considering the efforts made to isolate the complete sequence of all the previous reported *HpAOX* genes (Ferreira et al., 2009) it is here hypothesized that the *AOX* gene family in *H. perforatum* is composed by only two gene members.

Alternative oxidase is a diiron carboxylate protein composed by two monomers (dimeric protein). Each monomer is composed by six long helices (α1 to α6) and four short helices (αS1 to αS4) arranged in an antiparallel fashion. Helices α2, α3, α5, and α6 forming a four-helix bundle (Moore et al., 2013) accommodate the active site composed by the diiron center, four universally conserved glutamate residues, and two universally conserved histidine residues. The alignment made across deduced AOX sequences, including both *Hp*AOX sequences, shows not only the high conservation on the regions of the long helices, but also the complete conservation of the four glutamate (Glu-201, Glu-240, Glu-291, and Glu-342) and the two histidine (His-243 and His-345) residues.

Nevertheless, an exception can be seen at the *Hp*AOX2 sequence where the Ile-230 is substituted by a Val. This position corresponds to the Ile-207 in *Sauromatum guttatum* and to the Ile-152 in *Trypanosoma brucei* previously investigated by mutagenesis approach (see review at Moore et al., 2013). In an attempt to elucidate about the occurrence of this change, an alignment was constructed including high number of AOX sequences available at different Gene Banks (not shown). This allowed the identification of the same amino acid change in a sequence of *V. vinifera* (acc. VV00G00110). Nevertheless, no data are available about the effect of this mutation on the inhibition of respiration.

Besides the effect that those changes at gene sequence level could have on protein functionality (see review at Moore et al. (2013), post-transcriptional events have also a role on *AOX* regulation. In plants, *AOX* is controlled at post-transcriptional level by two interrelated mechanisms, being one of them dependent of α-keto acid regulation in which two cysteine residues are involved, CysI-145 and CysII-195 (Umbach et al., 2002), both conserved in *HpAOX* sequences. Nevertheless, some plant AOX lack the conserved CysI, having a SerI residue instead (Umbach and Siedow, 1993; Costa et al., 2009) which change the regulation from pyruvate to succinate (Holtzapffel et al., 2003; Grant et al., 2009). Recently, Moore et al. (2013) suggested that *AOX* regulation might also occur via phosphorylation of the N-terminal extension through charge-induced conformational changes and/or an interaction with other mitochondrial proteins. AOX N-terminal region is known as the less conserved region at size and sequence level, with the exception of the CysI, and has been considered independently from the structural defined four-helix bundle (Moore et al., 2013). The distance between CysI and CysII is 50 amino acids, as previously reported by Moore et al. (2013), but the distance between the predicted N-terminal sequence and CysI varies. This variation can be explained by the variability at genomic level observed at the level of exon 1 since the other exons show a conservation in size across genes with the four exons structure (exon 2: 129, exon 3: 489, exon 4: 57). The prediction of the mitochondrial signal peptide located at N-terminal region revealed no conservation at sequence and size level, going from very short mTP (AtAOX1b, acc. AT3G22360) to much longer (AtAOX2, acc. AT5G64210) although from the same plant species. This region determines the interaction of the peptide with the protein transport system to the organelle, and in many cases, amino acids comprising the signal peptide are cleaved off the protein once they reach their final destination. Finnegan et al. (1997) referred that proteins requiring N-terminal signals for mitochondrial import typically present a lack in overall sequence conservation at the N terminus. Huang et al. (2009) also reported a high variability in this region in a comparison between *Arabidopsis* and rice using a high set of proteins, describing that this region varied greatly from 19 to 109 amino acids in *Arabidopsis*, and from 18 to 117 amino acids in rice. Specifically related with AOX, Campos et al. (2009, 2016) described length variability at the mTP across plant species and also between carrot protein isoforms. More recently, Nogales et al. (2016) reported the presence of single nucleotide polymorphisms (SNPs) within the mTP sequence in *AOX1* sequences across carrot genotypes.

The effect of those differences in the N-terminal region for the regulation of gene expression, protein transport or activity is still unknown. Concerning the effect of the mTP sequence composition, structural studies revealed the importance of hydrophobic residues for mTP binding (Huang et al., 2009), and several studies on yeast, mammals, and plants revealed also that positively charged residues in mTP have a relevant role in protein import into mitochondria (Lister et al., 2005; Neupert and Herrmann, 2007). In plants, AOX are encoded by genes commonly composed by four exons interrupted by three size variable introns. Both *HpAOX* genes present the four exons structure, with the three last exons showing size conservation. Nevertheless, events of intron loss and gain, responsible by the variability in gene structure and consequently in exons size variation, were previously reported in *AOX* genes. A detailed description about *AOX* gene structure variation can be found in different reports (Polidoros et al., 2009; Cardoso et al., 2015; Campos et al., 2016).

In an attempt to search for putative motifs in *HpAOX* genes that could be further used to develop molecular markers, the intronic regions were explored *in silico*. Previous reports pointed out the existence of intron length polymorphism at individual plant level in *HpAOX1*, thus, pointing to the possibility for allelic discrimination (Ferreira et al., 2009). More recently Velada et al. (2014) pointed out the response of both *HpAOX* genes upon temperature stress. These observations are highly promising for a perspective toward FM development. Sequence variability at intron level in *AOX* genes has been suggested as a good source for functional polymorphisms that could be useful for FM development (Cardoso et al., 2009, 2011; Santos Macedo et al., 2009; Hedayati et al., 2015). In the present study, the identification of SSRs located at intronic regions of both *HpAOX* genes makes them genes of interest for further search for variability among genotypes that could be used for FM development. Besides its abundance, SSRs are highly polymorphic compared with other genetic markers, as well as being species-specific and co-dominant. For these reasons, they have become increasingly important genetic markers ideal for detecting differences between and within species of genes of all eukaryotes (Farooq and Azam, 2002). Nevertheless, variation in the length of microsatellite motifs in protein non-coding gene sequences (i.e., promoters, UTRs, and introns) may affect the process of transcription and translation through slippage, gene silencing and pre-mRNA splicing as has been observed for many human disorders (e.g., Kim et al., 2001), and also in some plant species (Tan and Zhang, 2001; Bao et al., 2006; Wang et al., 2011). Therefore, SSR markers generated from those sequence motifs can be of great use in terms of FM selection for genomic studies and crop breeding applications (Parida et al., 2009). However, the development of SSR markers located in potentially functional genic sequences in plants has been scarce, and the unique example known is in *Eucalyptus globules* for wood quality traits (Acuña et al., 2012). The identification of SSRs within *AOX* gene sequences was firstly reported by Nogales et al. (2016) with the recognition of TTCTT tandem repeats in intron 1 of carrot *AOX1* gene.

The increasing knowledge on the involvement of introns in the regulation of gene expression (Rose et al., 2008; Goebels et al., 2013; Heyn et al., 2015), particularly the encoding of important regulatory elements, makes introns of great interest for the identification of polymorphisms that can be responsible for phenotypical differences. MicroRNAs are an example of regulatory elements that can be encoded at intronic regions, playing its control by negatively regulating gene expression at post-transcriptional level. In plants, miRNAs play a critical role in almost all biological and metabolic processes, including plant development (Chen et al., 2015) and plant stress response (Bej and Basak, 2014). The identification of homologous miRNAs in distinct plant species is facilitated since many families of miRNAs are evolutionarily conserved across all principal lineages of plants (Axtell and Bartel, 2005; Zhang et al., 2006). Considering this knowledge, putative locations coding for precursors of miRNAs were identified at intronic regions of both *HpAOX* genes. In all cases, a high homology with previously described miRNAs was found.

In the same way, also transposable elements (TE), which includes intact TEs, degenerate TEs and sequence residues of mobile elements (TE remnants), can also influence the regulation of gene expression (McDonald, 1993; Brosius, 2000; Bowen and McDonald, 2001; Ganko et al., 2001; Nekrutenko and Li, 2001), which leads to a significant effect in functional genome diversity and phenotypic variations (Yadav et al., 2015). The *in silico* analysis searching for TEs allowed us to identify a 119 bp sequence in intron 1 of *HpAOX2* gene that showed similarity to the long terminal repeat region of a LTR retrotransposon (*Cassandra* MT-LTR) from *Medicago truncatula*. Whether this LTR remnant has any role in the regulation of *AOX* expression still needs to be elucidated, but the fact that several TEs and their evolved sequences are highly polymorphic in sequence and in genome location facilitates development of TE-based markers for various genotyping purposes.

### Analysis of transcript expression

The germination process involves many metabolic activities including a marked development of the photosynthetic and respiratory metabolisms. Thus, it is expected that AOX, like other antioxidative enzymes, would be involved in the process of seed germination and seedling development by the control of ROS produced during the germination process, and also in the promotion of the cellular homeostasis under the large metabolic changes that take place during germination and post-germination development.

To the best of our knowledge, this is the first report on the involvement of *AOX* genes in the post-germinative development of seedlings in *H. perforatum*. In the present study, a high increase for *HpAOX1* transcript was observed from 8 dps on. On the contrary, *HpAOX2* showed less variation in the expression of its transcript even if a slight tendency to decrease was observed right from day 4. These results are in agreement with other studies showing, for example, a decrease in the relative abundance of *AOX2* transcripts during seedling development in soybean, whereas the transcripts of the other *AOX* genes increased (McCabe et al., 1998). Other example are the findings from Saisho et al. (2001) demonstrating that the expression of *AOX2* in *Arabidopsis* was high in dry seeds and subsequently decreased during early germination, whereas *AOX1a* was less abundant at the beginning of the process, increasing only in a later stage. Several examples illustrate, as well, the involvement of *AOX* on plant vegetative growth and reproductive performance. In general, *AOX1* sub-family members are reported as more responsive genes upon stress factors, whereas *AOX2* members were considered during years not being affected by stress conditions, being more involved in developmental and growth processes events (Considine et al., 2002; Chai et al., 2012). Nevertheless, further studies have showed that *AOX2* members are also involved in plastid-dependent signaling (Clifton et al., 2005) and in plant stress response (Costa et al., 2010; Campos et al., 2016).

Interestingly, the increment in *HpAOX1* mRNA levels was accompanied by also a marked increase of the chloroplast *HpGAPA* transcript, having both transcripts a similar expression pattern during the post-germinative process of seedlings. *HpGAPA* was first used in our study in the expression stability analysis to select the most suitable RGs because it is commonly used as reference gene in data normalization of RT-qPCR studies, and particularly in studies involving gene expression during growth and development of seedlings, like in *Zea mays* (Sytykiewicz, 2014), *Arabidopsis thaliana* (Rigas et al., 2009), *Oryza sativa* (Ismail et al., 2009), or Triticum sp. (Boutrot et al., 2005). However, in our study, *HpGAPA* showed to be the most unstable gene among all tested candidate reference genes, and therefore, it seems not to be appropriate to be used as RG in the experimental conditions of the present study. Our findings are in accordance with other recent studies demonstrating also the unsuitability of this gene for data normalization purposes involving gene expression in seedlings, such as in *Sasha inchi* (Niu et al., 2015), or in *Oryza sativa* (Moraes et al., 2015). The gene product of *GAPA* is a key enzyme in the photosynthetic carbon fixation pathway (Cerff, 1982), and therefore, the marked increase of its transcript observed in our study might indicate an increment of the photosynthetic activity during the post-germinative process. In fact, green tissues started to be more apparent at that time (Figure 1). Dewdney et al. (1993) found that the expression of the *GAPA* gene is regulated by light in *Arabidopsis* seedlings. In the same line, other works showed that the increase of total photosynthetic activity was accompanied by an increase in the GAPDH activity in response to light (Shih and Goodman, 1988 and references therein).

Taken together, it could be hypothesized that the increment in *GAPA* gene expression, and possibly in the photosynthetic activity, is closely related to an increased need of the *HpAOX1* enzyme during the post-germinative process of *H. perforatum*. This would be in accordance with previous reports about the role of AOX in optimizing photosynthesis (Dinakar et al., 2016). Indeed, it has been reported that AOX has a role in optimizing photosynthesis, e.g., by maintaining a balanced carbon and energy metabolisms (Watanabe et al., 2008), by keeping up the light activation of chloroplastic enzymes (Padmasree and Raghavendra, 2001), by functioning as a sink for the excess reducing equivalents generated by photosynthesis (Yoshida et al., 2006, 2007), or by regulating ROS levels (Dinakar et al., 2016). With regard to the latter, during active photosynthesis there are four major types of ROS being produced in green tissues, such as, singlet oxygen (O_2_), superoxide (${\text{O}}_{2}^{-}$), hydrogen peroxide (H_2_O_2_), and hydroxyl radicals (OH^−^) (Apel and Hirt, 2004). AOX has been described as a potential means to dampen ${\text{O}}_{2}^{-}$ production by the ETC (Purvis and Shewfelt, 1993) and catalase, other antioxidative enzyme also analyzed in our study, as having a role in the hydrogen peroxide scavenging (Puntarulo et al., 1988; Møller, 2001; Mittler, 2002; Dinakar et al., 2010).

Unlike *HpAOX1* and *HpGAPA, HpCAT1* revealed a gradual down-regulation in its transcript levels reaching the peak of down-regulation at 12 dps, which suggests an involvement of *HpCAT1* during the post-germinative process in *H. perforatum*. Several studies imply that CAT activity is necessary for seed germination and early seedling growth and its measurement may be used as a parameter to examine seed viability and germination (Ak et al., 2012 and references therein). Indeed, most of these works revealed higher levels of catalase activity related to germination, whereas our data revealed a down-regulated expression for this gene. It should not rule out the possibility that other catalase isoforms may be present in this process, having a different behavior. However, they were not included in this study since no information about their nucleotide sequence are available in databases. Additionally, it must be noticed that in our study the germination process has already finished at the first time point considered (4 dps), since the radicle emerging from the seed coat was already observable. The subsequent steps consisted in the post-germinative process with the development of seedlings. Therefore, it would be plausible that the peak observed on *HpCAT1* expression might have occurred earlier during the first stages of *H. perforatum* seeds germination (before 4 dps), when *HpCAT1* is more required, and that the subsequent decrease on transcript accumulation that we observed might be related to the necessity of maintaining ROS at adequate levels for cellular survival. Indeed, it has been reported that excessive removal of hydrogen peroxide free radicals might reduce the inhibition of the cellular cycle, which is important for DNA repair (Santos et al., 2013). In the same line, cellular biology studies have clearly described the beneficial functions of hydrogen peroxide to the cell, beyond causing oxidative stress. One of these beneficial functions is stopping the cellular cycle during DNA repair after certain types of stress and aging (Santos et al., 2013). Accordingly to our results, Mhamdi et al. (2012) reported that a transcriptional down-regulation of *CAT* could be important to induce or sustain increased H_2_O_2_ availability necessary for certain environmental responses or developmental processes.

Taken together, we suggest that fluctuations in the expression between *HpAOX1* and *HpCAT1* might occur in order to control ROS levels during the germination process in *H. perforatum*.

In summary, to the best of our knowledge, this is the first study in which the characterization of the *AOX* gene family is reported in *H. perforatum* and the expression of their transcripts are analyzed during the post-germinative development of seedlings. Two *HpAOX* genes were identified, one belonging to the *AOX1*-subfamily and another to the *AOX2*-subfamily. Sequence variability was observed at 3′ and 5′ ends. At the N-terminal region, the variability was reported across *AOX* gene members within a species and among species. Besides, SSR, a TE remnant and putative miRNA coding sequences were detected in intronic regions of *HpAOX* genes, whose variability could be explored across genotypes to identify polymorphisms with functional significance that would allow functional marker development. Our findings, although preliminary, lead us to consider *HpAOX* members, in particular *HpAOX1*, as relevant to investigate further, also at the protein level (e.g., amount, activity and capacity), in post-germinative processes in order to explore its role in optimizing photosynthesis and in the control of ROS levels. This would help making the link between gene function and the desired phenotype related to better germination rates and consequently to develop a functional marker for it.

## Author contributions

HC contributed to the conception and design of the work; to the acquisition, analysis, and interpretation of data; to the drafting and critical revision of the work; and to the final approval of the manuscript; IV contributed to the acquisition, analysis, and interpretation of data; to the drafting and critical revision of the work, and to the final approval of the manuscript; CR contributed to the conception and design of the work; to the acquisition of data; to the drafting and revision of the work; and to the final approval of the manuscript; AN contributed to the analysis and interpretation of data; to the drafting and critical revision of the work; and to the final approval of the manuscript; AF contributed to the conception and design of the work; to the acquisition of data; to the drafting of the work and to the final approval of the manuscript; VV contributed to the acquisition of data; to the drafting of the work; and to the final approval of the manuscript; BA contributed to the interpretation of data; to the critical revision of the work; and to the final approval of the manuscript. All authors are responsible for all the work.

## Funding

This work was funded by the European Commission (MEXC-CT-2004-006669) through providing the EU Marie Curie Chair and by the National Funds through FCT - Foundation for Science and Technology under the Project UID/AGR/00115/2013, and under the project PTDC/AGRGPL/099263/2008. The authors are also thankful to FCT for the support provided under the program POPH-Programa Operacional Potencial Humano to BA and HC (Ciência 2007 and Ciência 2008: C2008-UE/ICAM/06), and also to ICAAM and UEvora for the support given to HC (BPD UEvora ICAAM INCENTIVO AGR UI0115 and BI_PosDoc_UEVORA_Calorespirometria_2).

### Conflict of interest statement

The authors declare that the research was conducted in the absence of any commercial or financial relationships that could be construed as a potential conflict of interest.
