# Supplementary material for: Alternative Oxidase Gene Family in Hypericum perforatum L.: Characterization and Expression at the Post-germinative Phase
Source: Front Plant Sci. 2016 Aug 11;7:1043. doi: 10.3389/fpls.2016.01043 (PMC4980395; doi:10.3389/fpls.2016.01043)
Supplement: Supplementary file 1 [file DataSheet1.pdf]

Supplementary Material

Alternative oxidase gene family in *Hypericum perforatum* L.: characterization and expression at the post-germinative phase

Isabel Velada[1†], Hélia G. Cardoso[1†*], Carla Ragonezi[1], Amaia Nogales[2], Alexandre Ferreira[1], Vera Valadas[1] and Birgit Arnholdt-Schmitt1*

[1]EU Marie Curie Chair, ICAAM - Instituto de Ciências Agrárias e Ambientais Mediterrânicas, Universidade de Évora, Pólo da Mitra, Ap. 94, 7002-554 Évora, Portugal, [2]Linking Landscape, Environment, Agriculture and Food, Instituto Superior de Agronomia-Universidade de Lisboa, Lisboa, Portugal

[†]These authors have contributed equally to this work.

*** Correspondence:** Corresponding Authors: eu_chair@uevora.pt and hcardoso@uevora.pt

1 Supplementary Figures and Tables

1.1 Supplementary Figures

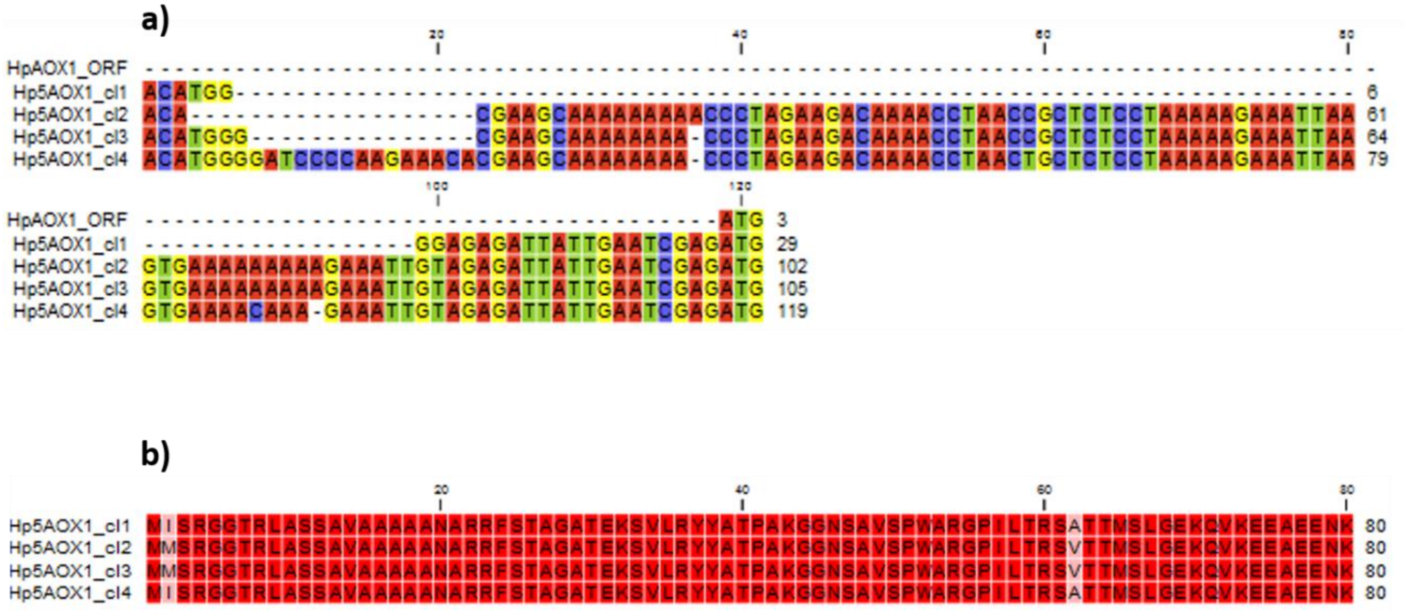

Figure S1. Alignment of the four isolated sequences corresponding to 5'end of *HpAOX1* gene. The sequences correspond to the region from the primer adapter until ATG codon (included in the alignment). **a)** Alignment of the nucleotide sequences; **b)** Alignment of the corresponding putative translated peptide where no alternative ATG codon was identified.

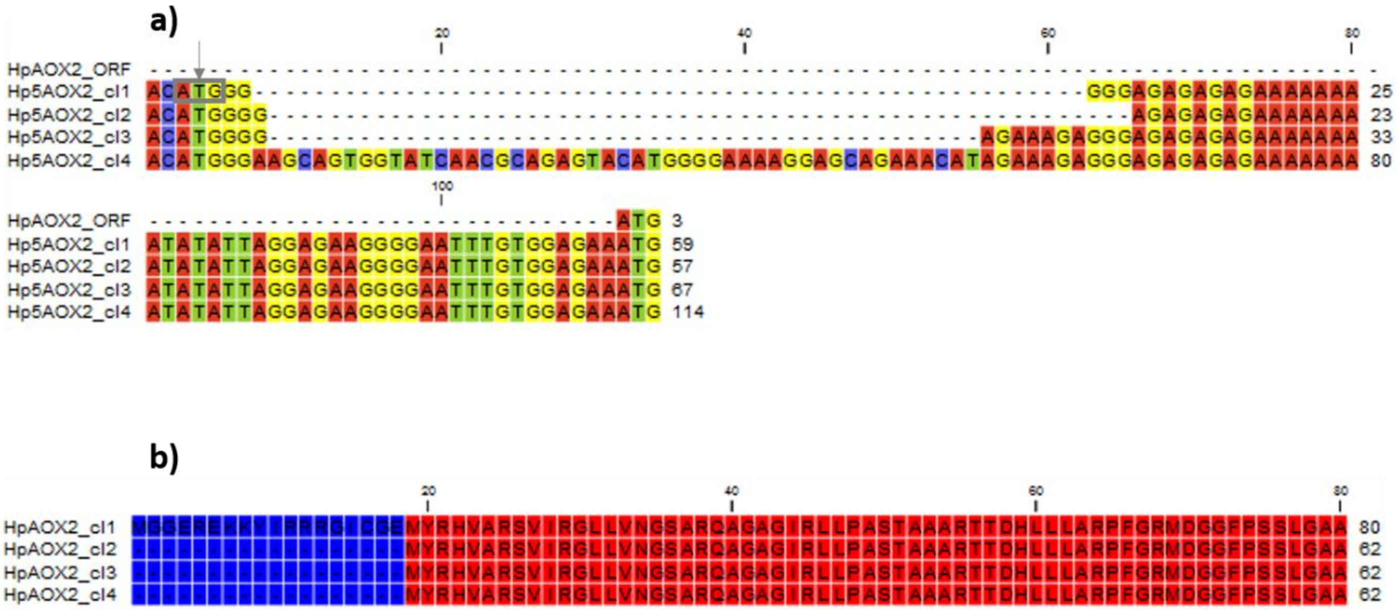

Figure S2. Alignment of the four isolated sequences corresponding to 5'end of *HpAOX2* gene. The sequences correspond to the region from the primer adapter until ATG codon (included in the alignment). **a)** Alignment of the nucleotide sequences; **b)** Alignment of the corresponding putative translated peptide where an alternative ATG codon was identified in the sequence HpAOX2_cl1 (region in blue).

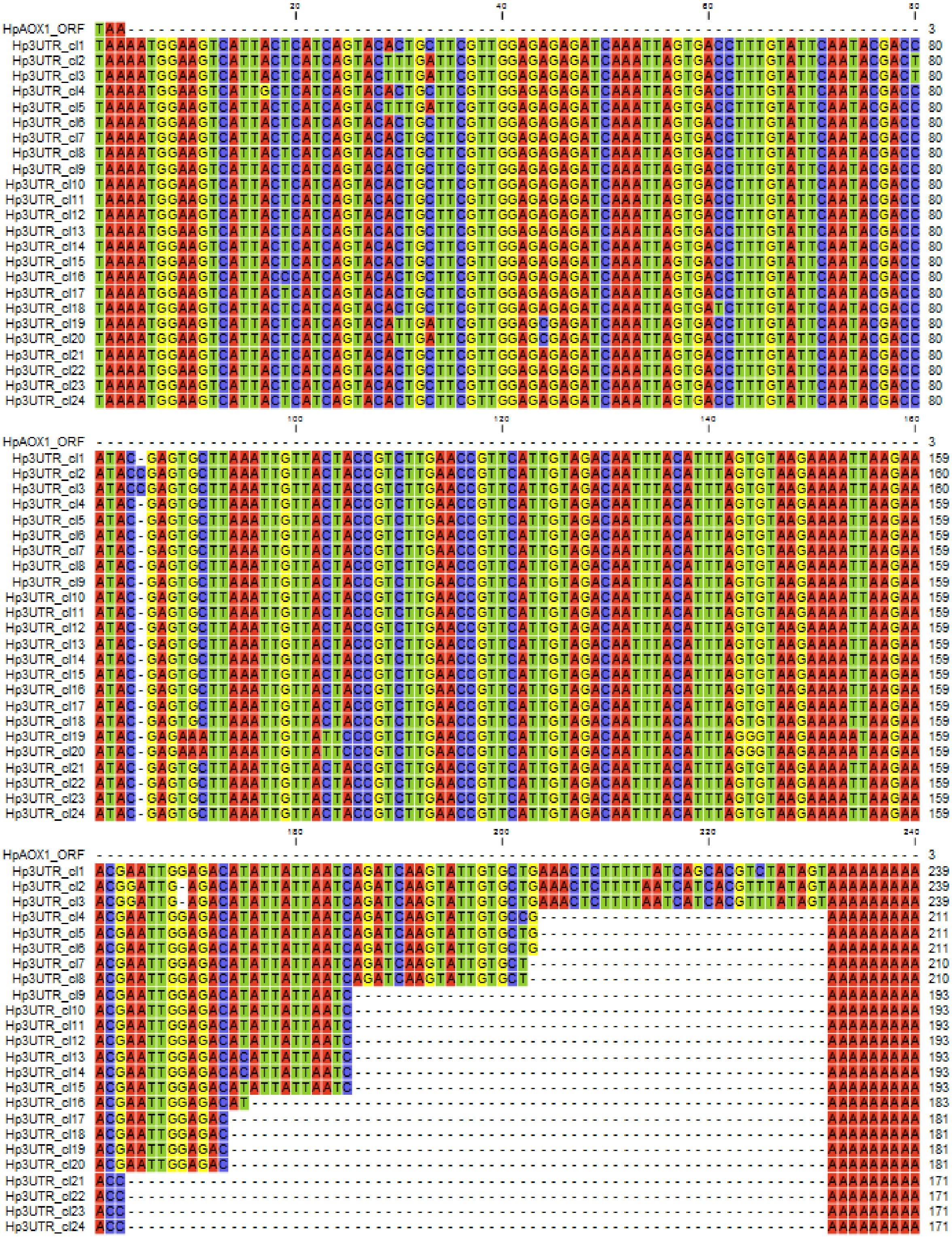

Figure S3. Alignment of the twenty four isolated sequences corresponding to 3'end of *HpAOX1* gene. The sequences correspond to the region from the stop codon (TAA, included in the alignment) to the poly(A)-tail (only nine common adenines are represented).

ACATGGGGATCCCCAAGAAACACGAAGCAAAAAAAACCCTAGAAGACAAAACCTAACTGCTCTCCTAAAAAG
AAATTAAG 80
TGAAAACAAAGAAATTGTAGAGATTATTGAATCGAGATGATAAGCCGCGGCGGTACTAGGCTGGCGAGTTCG
GCGGTTGC 160
M I S R G G T R L A S S A V A

GGCGGCCGCTGCCAATGCTCGGCGCTTCTCGACTGCCGGAGCAACGGAGAAGTCTGTTCTCAGATACTATGC
TACTCCGG 240
A A A A N A R R F S T A G A T E K S V L R Y Y A T P

CAAAGGGCGGAAACAGTGCGGTGTCGCCGTGGGCGAGGGGCCCGATCTTGACGAGGAGTGCAACGACGATG
AGCTTGGGA 320
A K G G N S A V S P W A R G P I L T R S A T T M S L G

GAGAAGCAAGTGAAGGAGGAGGCTGAAGAGAATAAGGTTCCTTCGGAGCGTTCCACCGGCGCCGGAGGGAA
CAAGGATGG 400
E K Q V K E E A E E N K V P S E R S T G A G G N K D G

CGGCGATGAGAAGGCCGTTGTTAGCTACTGGGGTATTAACCCCGGGAAGATCACTAAGGAAGACGGCACCGT
TTGGACCT 480
G D E K A V V S Y W G I N P G K I T K E D G T V W T

GGACTTGCTTTAGGCCATGGGAAGCATACCAACCAAACATGTCGATTGATCTGAAGAAGCATCACGCTCCGA
CTACTTTC 560
W T C F R P W E A Y Q P N M S I D L K K H H A P T T F

▼
TTGGACAAACTGGCTTTTCGGACCGTTAAGTCTCTCCGATGGCCCACCGACATATTCTTTCAGAGGAGGTATG
GTTGTCG 640
L D K L A F R T V K S L R W P T D I F F Q R R Y G C R

GGCAATGATGCTCGAAACAGTGGCCGCGGTGCCAGGCATGGTAGGAGGAATGCTCCTCCACTGCAAGTCACT
GAGGCGCT 720
A M M L E T V A A V P G M V G G M L L H C K S L R R

TCGAGCACAGCGGAGGATGGATCAAAACCCTACTGGACGAGGCAGAGAACGAGCGGATGCACTTGATGACA
TTCATGGAG 800
F E H S G G W I K T L L D E A E N E R M H L M T F M E

GTTGCCAAGCCCAAATGGTACGAGCGTGCTCTGGTCTTCACAGTGCAGGGCGCCTTCTTCAACATCTACTTTC
TGGGGTA 880
V A K P K W Y E R A L V F T V Q G A F F N I Y F L G Y

TCTCATTTCCCCCAAGTTCGCGCATAGGGTGGTTGGATACCTCGAGGAAGAAGCAATCCACTCCTACACCGA
GTTTCTCA 960
▼
L I S P K F A H R V V G Y L E E E A I H S Y T E F L

AGGAGTTGGACAATGGCAACATCGAGAATGTGCCTGCTCCTGCAATTGCTGTCGACTACTGGCGCCTACCTC
CCAACTCC 1040
K E L D N G N I E N V P A P A I A V D Y W R L P P N S

ACTCTAAAGGATGTGGTTCAGGTTGTCAGGGCCGACGAGGCTCATCACCGCGACGTCAATCATTTTGCATCG
GACATACA 1120
T L K D V V Q V V R A D E A H H R D V N H F A S D I H

CTACCAAGGACGTGAGCTGAGGGAATGCCCTGCTCCACTGGGTTATCACTAAAATGGAAGTCATTGCTCATC
AGTACACT 1200

Y Q G R E L R E C P A P L G Y H *

GCTTCGTTGGAGAGAGATCAAATTAGTGACCTTTGTATTCAATACGACCATACGAGTGCTTAAATTGTTACTA
CCGTCTT 1280
GAACCGTTCATTGTAGACAATTTACATTTAGTGTAAGAAAATTAAGAAACGAATTGGAGACATATTATTAATC
AGATCAA 1360
GTATTGTGCTGAAACTCTTTTAATCATCACGTTTATAGTAAAAAAAAAAAAAAAA 1415

Figure S4. Nucleotide and deduced amino acid sequences of cDNA encoding *Hypericum perforatum* L. alternative oxidase 1 (*HpAOX1*, acc. no. EU330415.1). ▼ indicates the position of the three introns, * indicates stop codon.

ACATGGGAAGCAGTGGTATCAACGCAGAGTACATGGGGAAAAGGAGCAGAAACATAGAAAGAGGGAGAGAG
AGAAAAAAA 80
ATATATTAGGAGAAGGGGAATTTGTGGAGAAATGTACCGCCACGTGGCAAGGTCAGTGATCAGGGGACTCCT
GGTCAACG 160
M Y R H V A R S V I R G L L V N

GGAGTGCCCGCCAGGCTGGCGCCGGGATCCGGCTGCTTCCAGCTTCCACAGCCGCCGCGGACGACCGAC
CACCTCCTC 240
G S A R Q A G A G I R L L P A S T A A A R T T D H L L

CTCGCGAGGCCTTTCGGCCGGATGGACGGTGGATTCCCGTCCTCGCTCGGCGCGGCGAGTTACTTGAGGATG
TTGAGCAG 320
L A R P F G R M D G G F P S S L G A A S Y L R M L S S

CTCATCGTCGTCGGAGGGGAAGCCGGTGGCGGAGGAGGAGAAGAAGGCGGTGGCGTCCAGTTACTGGGGAA
TCTCGAGGC 400
S S S S E G K P V A E E E K K A V A S S Y W G I S R

▼
CGAAGGTCACTAGGGAGGACGGCACCGACTGGCCTTGGAACTGCTTCATGCCTTGGGAGTCTTATAGGACAG
ATACGTCA 480
P K V T R E D G T D W P W N C F M P W E S Y R T D T S

ATAGATCTGACGAAGCACCACGTTCCAAAGACATTTCTTGACAAGTTTGCTTACTGGACGGTCAAGATCCTTC
GAATTCC 560
I D L T K H H V P K T F L D K F A Y W T V K I L R I P

▼
TACAGATATATTCTTTCAGAGACGGTACGGCTGCCGCGCAATGATGTTGGAAACAGTTGCAGCTGTACCGGG
TATGGTTG 640
T D I F F Q R R Y G C R A M M L E T V A A V P G M V

GTGGGATGCTTCTGCACTTGAGGTCGCTCCGCAAGTTCGAGCAAACTGGGGGTTGGGTCAAGGCCTTGCTCG
AAGAAGCA 720
G G M L L H L R S L R K F E Q T G G W V K A L L E E A

GAGAACGAAAGAATGCACTTGATGACAATGGTAGAGCTCGTTCAGCCAAAATGGTACGAGAGGATGCTGGTT
CTTATCGT 800
E N E R M H L M T M V E L V Q P K W Y E R M L V L I V

GCAGGGGCTGTTCTTCAACGCCTACTTTGTGATCTATCTCGTGTCCCCGAAGCTCGCTCACAGGGTAACAGG
CTATTTGG 880
Q G L F F N A Y F V I Y L V S P K L A H R V T G Y L

AAGAAGAGGCCATTCATTCATATACCGAGTACCTCAAAGACATAAGGAGTGGGAAAATCGAGAATGTCAAGG
CTCCCGCT 960
E E E A I H S Y T E Y L K D I R S G K I E N V K A P A

ATTGCGATCGACTACTGGAGGTTGCCTAAGGAAGCTACACTCGAGGATGTCATAACTGTGATCCGAGCCGAT
GAAGCACA 1040
I A I D Y W R L P K E A T L E D V I T V I R A D E A H

▼
TCACCGTGATGTCAACCACTTTGCTTCTGATGTTCACTATCAGGGCAAGAAACTGAAAGAGGCACCTGCTCCT
ATTGGCT 1120
H R D V N H F A S D V H Y Q G K K L K E A P A P I G

ATCATTGAGGGGTTTTTTTTTTTTTTTAACTTATGTTTTCAGAGTCAAGTAATATCGAAATTTATTCGAGACTT
ATTTAG 1200
Y H *

TTCTTACTTCAGCTTGTGTTGTATATGCTATGTATTGTAAACGTATATTATTGTAAAATATAAGGCGCATATAA TATACA 1280
TTGTTTTGTTGACATAAAAAAAAAAAAAAAA 1311

Figure S5. Nucleotide and deduced amino acid sequences of cDNA encoding *Hypericum perforatum* L. alternative oxidase 2 (*HpAOX2*, acc. no. EU330413.1). ▼ indicates the position of the three introns, * indicates stop codon.

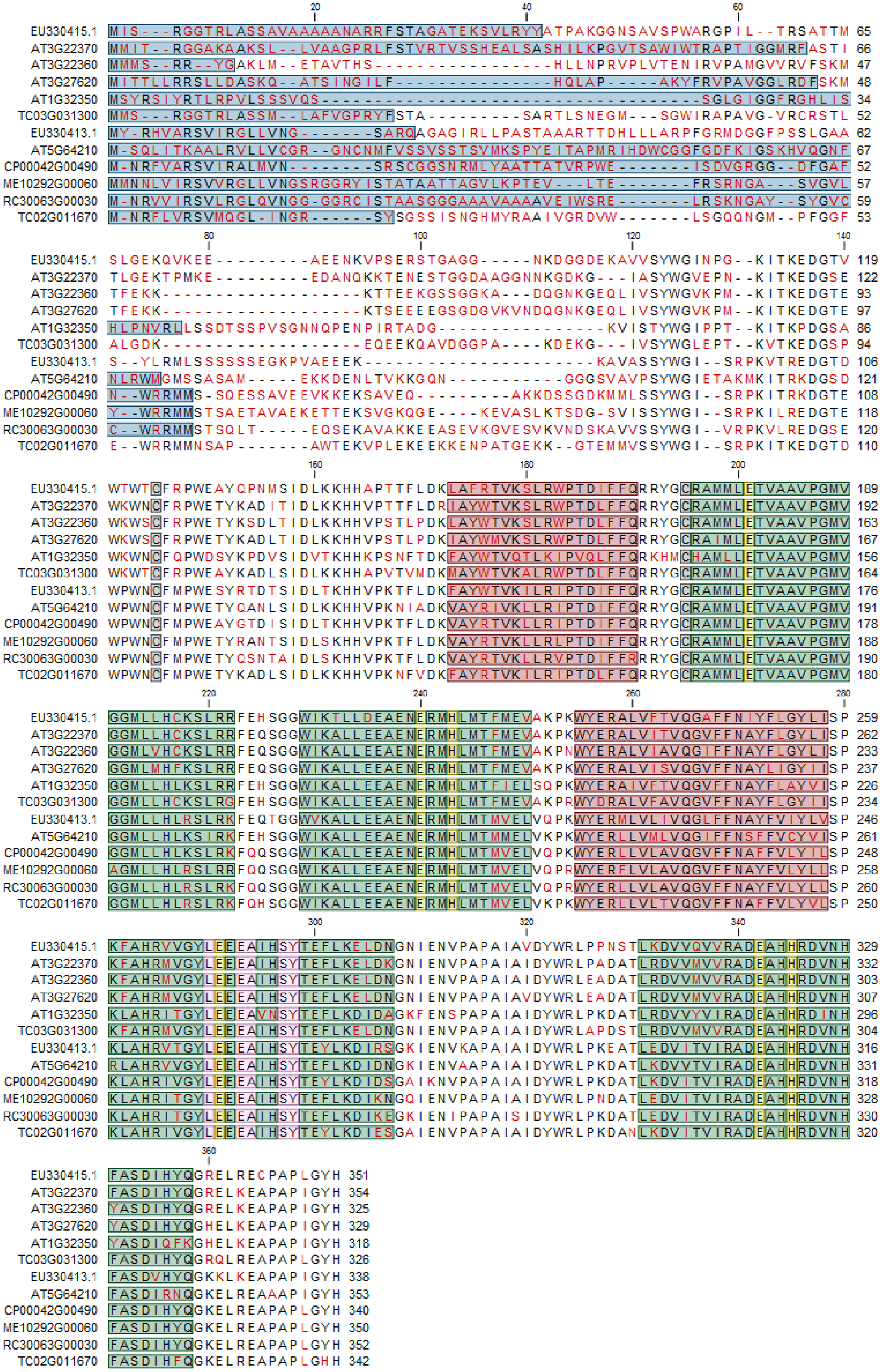
EU330415.1
AT3G22370
AT3G22360
AT3G27620
AT1G32350
TC03G031300
EU330413.1
AT5G64210
CP00042G00490
ME10292G00060
RC30063G00030
TC02G011670

Figure S6. Multiple alignment of translated amino acid sequences of previously reported AOX proteins from, *Arabidopsis thaliana* (AtAOX1a_AT3G22370, AtAOX1b_AT3G22360, AtAOX1c_AT3G27620, AtAOX1d_AT1G32350 and AtAOX2_AT5G64210), *Theobroma cacao* (TcAOX1_TC03G031300 and TcAOX2_TC02G011670) *Carica papaya* (CpAOX2_CP00042G00490), *Manihot esculenta* (MeAOX2_ME10292G00060), *Ricinus communis* (RcAOX2_RC30063G00030) and AOX from *Hypericum perforatum* (HpAOX1_EU330415.2 and HpAOX2_EU330413.2). The alignment was performed using CLC Main Workbench 6.7.1 software. The data were retrieved from public web-based database Plaza v2.5, freely available at http://bioinformatics.psb.ugent.be/plaza/versions/plaza_v2_5/. Amino acids residues differing are shown in red, deletions are shown by minus signs. The putative mitochondrial transit peptides (mTP) are shown in blue boxes. The sites of two conserved cysteins (CysI and CysII) involved in dimerization of the AOX protein by S–S bond formation (Umbach and Siedow, 1993) are indicated in dark grey boxes. Helices α1 and α4, which form the hydrophobic region on the AOX molecular surface and are involved in membrane binding, are shown in red; helices α2, α3, α5 and α6, which form the four-helix bundle accommodating the diiron center, are shown in green (Moore et al. 2013). Amino acids residues that coordinate the diiron center (E, glutamate and H, histidine) and those that interact with the inhibitor are in yellow and light pink boxes, respectively.

ATGATAAGCCGCGGCGGTACTAGGCTGGCGAGTTCGGCGGTTGCGGCGGCCGCTGCCAATGCTCGGTGCT
TCTCGACGGCCGGAGCCACGGAGAGGTCTGTTCTCAGATACCATGCTACTCCGGCGAAGGGCGGAAACAG
TGCGGTGTCGCCGTGGGCGAGGGGCCCGATCTTGACGAGGAGTGTAACGACGATGAGCTTGGGAGAGAAG
CAAGTGAAGGAGGAGGCTGAAGAGAATAAGGTTCCTTCGGAGCGTTCCACCGGCGCCGGAGGGAACAAGG
ATGGCGGCGATGAGAAGGCCGTTGTTAGCTACTGGGGTATTAACCCCGGGAAGATCACTAAGGAAGACGG
CACCGTTTGGACGTGGATCTGTTTTAGGGTAAGCTTGATTTTATTTTATTTTTTCTTATTTTTTTTTGGT
AGTAATATTTTATTTTTTTTTCTTATTTCTTGACCTTCGTCAATAGAGATTTTGACTTCCTTTTGTCACGC
GGCGTTTGAATCTTTGACTTTGTTATTTAGGGATTTTTTTTGTTGTGGATTAATTTATATTTAATTGATAT
GATTTAGATGTTCTAATATATTAGGGAATTTTTGTTTTTGTGATGTATATTTAATTAAAATGTATTGGTA
AATGTGAAGCCATGGGAAGCATACCAACCAAACATGTCGATTGATCTGAAGAAGCATCACGCTCCGACTA
CTTTCTTGGACAAACTGGCTTTTTGGACCGTTAAGTCTCTCCGATGGCCCACCGACATATTCTTTCAGGT
AATATTTAATTTCTTCAATATTATTTTTTCGCATATCTTCTTCTAGGTTGAGATTACTACTGGGAGACTT
TTTTACTGAGAAAAATCTAAATAACATTGTTATAAAATTTTGGAGTGAACATGAAAATTTGAGTTTGGTA
TATCTTGGACATCGGTAGTATGTTCACATTTGTGACTTATTCAATGTTACAATTACCATCAAAAAAAAAA
TTAGGGTAAATTTCACTTGGTCATTGACCAGATTGTATAAAGAGACTCTAACACTACAATATGTATGTAT
ATATTGAATAATATATATATTTTCAACCCAACAAAATATATTTGAACCCTTCTATAATTTGTTGTGTTGC
ATCTGACGCCATTTTCCTGGTTCTGTTTTTTTTTTGGTGTAATCCTGGTTCTGTTGTTGTTTACTAACTT
TCCCCCAAAAAATTGTAATGTGAATTAATGAGAAAAATTCTCGTTAAGTCATCGAAAGAAGGATTTTTTT
TTAAATCTTCCTTCAAGAGGAGATCTATATTATTAGAAAGAGGAGGACAATGGGTGGCAGCTTAATTGGT
TCCTGGCGCCACCTTTTCTTGCCCTCTTTTACGTGAATCACTTGTCCATCAGTTGAGAACAGAAGGCTTT
GCTGTACCATGTAAGTCTCTAGTTAATGCAAAAAATTCGAGAAACTTTGGGTTGAATATGAAACCCAAGT
TATACTTAGATGAGAATCTGGTTTATTGTTTGTGTTTTATCACAAACTCTATGGACTTAAAAGACTTCCA
TTTAGAGTATGATTGTAGATAATTCTCTTCTGATTAATCTCTGGCATTATGTCATGCAGAGGAGGTATGG
TTGTCGGGCAATGATGCTCGAAACAGTGGCCGCGGTGCCAGGCATGGTAGGAGGAATGCTCCTCCACTGC
AAGTCACTGAGGCGCTTCGAGCACAGCGGAGGATGGATCAAAACCCTACTGGACGAGGCAGAGAACGAGC
GGATGCACTTGATGACATTCATGGAGGTTGCCAAGCCCAAATGGTACGAGCGTGCTCTGGTCTTCACAGT
GCAGGGCGCCTTCTTCAACATCTACTTTCTGGGGTATCTCATTTCCCCCAAGTTCGCGCATAGGGTGGTT
GGATACCTCGAGGAAGAAGCAATCCACTCCTACACCGAGTTTCTCAAGGAGTTGGACAATGGCAACATCG
AGAATGTGCCTGCTCCTGCAATTGCTGTCGACTACTGGCGCCTACCTCCCAACTCCACTCTAAAGGATGT
GGTTCAGGTTGTCAGGGCCGACGAGGCTCATCACCGCGACGTCAATCATTTTGCATCGGTAAATCAAGTT
CCCTTGTTTTGTTTTCCCCTAACAACCTGTGGTTTCTGCAGATTCTCGATGTGATACAAACGGTTGATAC
TATCGTCTTAAATGCAGGACATACACTACCAAGGACGTGAGCTGAGGGAATGCCCTGCTCCACTGGGTTA
TCACTAA

Figure S7. Genomic sequence of *Hypericum perforatum* L. alternative oxidase 1 (HpAOX1, acc. no. KU674355). Exons are in yellow, stop codon in pink, repetitive sequences in red, pre-miRNA sequences in grey. In blue there is the second predicted pre-miRNA sequence which is two base pairs shorter and was predicted as in reverse sequence.

ATGTACCGCCACGTGGCAAGGTCAGTGATCAGGGGACTCCTGGTCAACGGGAGTGCCCGCCAGGCTGGCG
CCGGGATCCGGCTGCTTCCAGCTTCCACAGCCGCCGCGCGGACGACCGACCACCTCCTCCTCGCGAGGCC
TTTCGGCCGGATGGACGGTGGATTCCCGTCCTCGCTCGGCGCGGCGAGTTACTTGAGGATGTTGAGCAGC
TCATCGTCGTCGGAGGGGAAGCCGGTGGCGGAGGAGGAGAAGAAGGCGGTGGCGTCCAGTTACTGGGGAA
TCTCGAGGCCGAAGGTCACTAGGGAGGACGGCACCGACTGGCCTTGGAACTGCTTCATGGTATTAACATA
TCTTAGAAAAGCTTGATACGATGTGATTAAATGATTTGATTGTTATTATCGATATTAACAAGTTTAGCGT
GGTTAGCCTCGGATAATTTTAGAATGGATAATCTGAGAAGTTGTTAGGGTGTGTGAACGAGGACAAAACA
CGCTGGAAAAAAGTTATCTAGATTTGTGTAGACACTCGGTCAGATCGCGGGATGTTACAGTATTAATAGT
AGAAATTTGAATTACATGCTACAAGCTTTACATGTTGTTTCGTTACGCAGTAGATCAGGTTCATAGCAGA
ATGCAGAAATCTTATTAGTTCAATTCCGTGCTTGTATGAATAACGCATGCGATCAGCCGGACAATTATAT
TTCGAGTTCAACAAGTATGGGAATGATGAAGTCTGTACTAGTTCAAAACTGCTTTATCAGATGTTAAACA
ACTTTTTTAGTTACGTGGAAATTTTCTTATTAGAAATGAACGAATTAAGTACATGGATGTGGATTTCTTT
CATTGTCTGAGACAGTTGGCCTACGATCTTGACGAGTCCTAGAGTATCGGGTTTGTCCTAAATCTTTTGT
GGCTACTTTGCCTGCTAAACCAATATGTAGCTTATATGAATTGCTCGTTTAACTAAGTGTATGTGTTTTG
TGGGGAACTTTGATTAAGAAGTGGAGTTGATTGAAGTTATGTTTATGGGTGTTGGTAGCCTTGGGAGTCT
TATAGGACAGATACGTCAATAGATCTGACGAAGCACCACGTTCCAAAGACATTTCTTGACAAGTTTGCTT
ACTGGACGGTCAAGATCCTTCGAATTCCTACAGATATATTCTTTCAGGTCAGGTTTGTTTAATCTTCATG
GGTTGTTCAAAATTTGGCATGAGTATTGGTGGTCTGGATAATCAATACAGAGCAAAGACTGTTTTCGATG
ATTGTCGTTAACTTGTGCCGCTGAATTAGTTTTGATCATCCACTGCAAAGTTTACTACACTTTGTTCCTC
TTGTCAAAGTTTTCACAAATCTTGTGAATCGTGTCTCAGAGACGGTACGGCTGCCGCGCAATGATGTTGG
AAACAGTTGCAGCTGTACCGGGTATGGTTGGTGGGATGCTTCTGCACCTGAGGTCACTCCGCAAGTTCGA
GCAAACCGGGGGTTGGGTCAAGGCCTTGCTCGAAGAAGCCGAGAACGAAAGAATGCACTTGATGACAATG
GTAGAGCTCGTTCAGCCAAAGTGGTACGAGAGGATGCTGGTTCTTATCGTGCAGGGGCTGTTCTTCAACG
CCTACTTTGTGATCTATCTCGTGTCCCCGAAGCTCGCTCACAGGGTAACAGGCTATTTGGAAGAAGAGGC
CATTCATTCATATACCGAGTACCTCAAAGACATAAGGAGTGGGAAAATCGAGAATGTCAAGGCTCCCGCT
ATTGCGATCGACTACTGGAGGTTGCCTAAGGAAGCTACACTCGAGGATGTCATAACTGTGATCCGAGCCG
ATGAAGCACATCACCGTGATGTCAACCACTTTGCTTCTGTAAGTATTAGCTCTCAATCTGAATTTATATT
TGGCATGCCAGTTACGAGATCGATAAGATTGATATACCAAACTTTCTTCGTGAACCAGTATTTGTTGTAA
AAAGCGATTCTTTGATTAAACAGAATTCATTTCAGATGGAATTCGATTTTACTCAATTCTACTCGGTCTT
TGGGTGCAGGATGTTCACTATCAGGGCAAGAAACTGAAAGAGGCACCTGCTCCTATTGGCTATCATTGA

Figure S8. Genomic sequence of HpAOX2 (acc. no. KU674356). Exons are in yellow, stop codon in pink, repetitive sequences in red, predicted LTR region homologous of a *Cassandra* MT-LTR retrotransposon sequence in green.

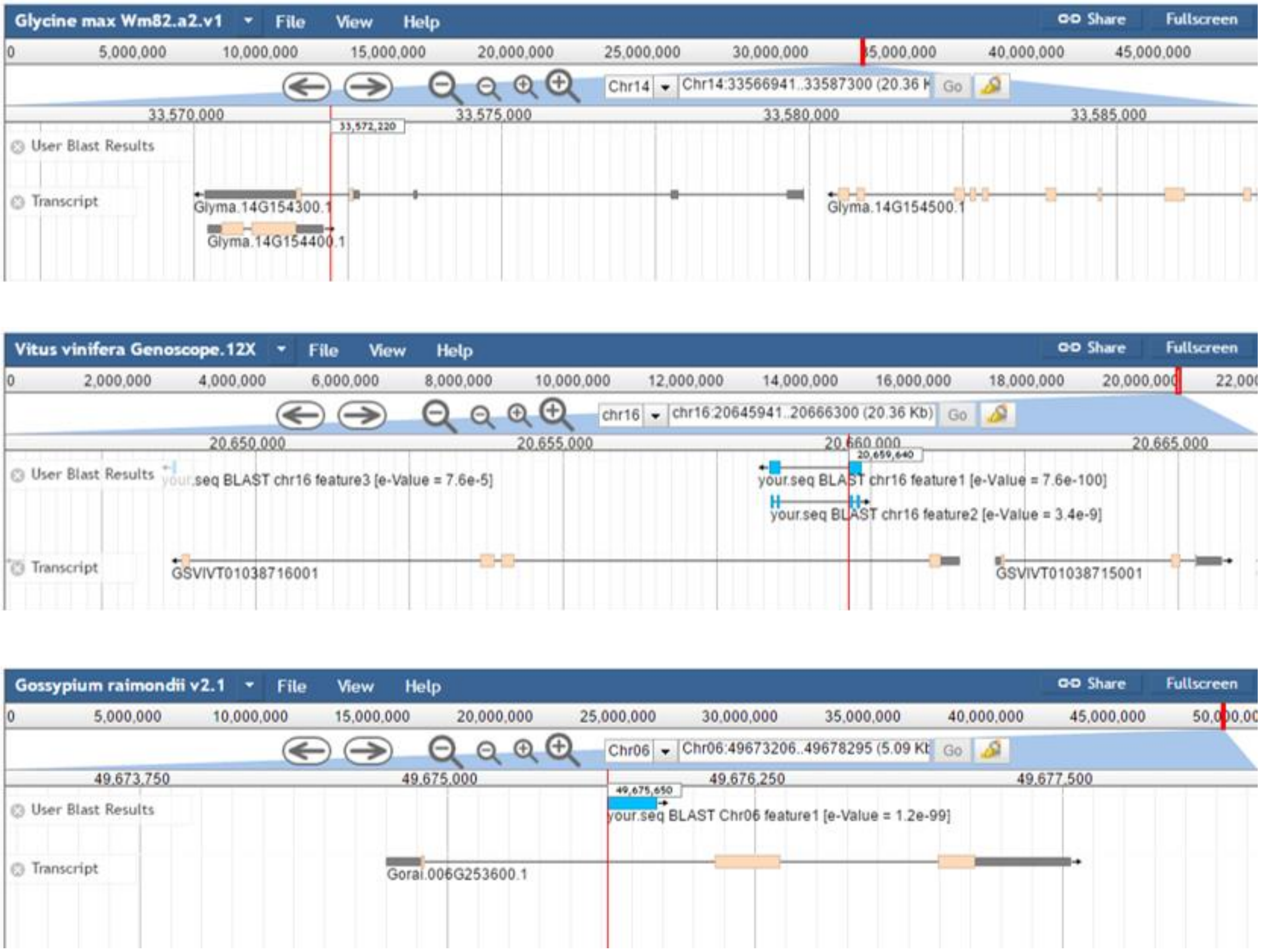

Figure S9. Genome mapping showing the location of the pre-miRNA sequences that encode the homologous miRNAs.

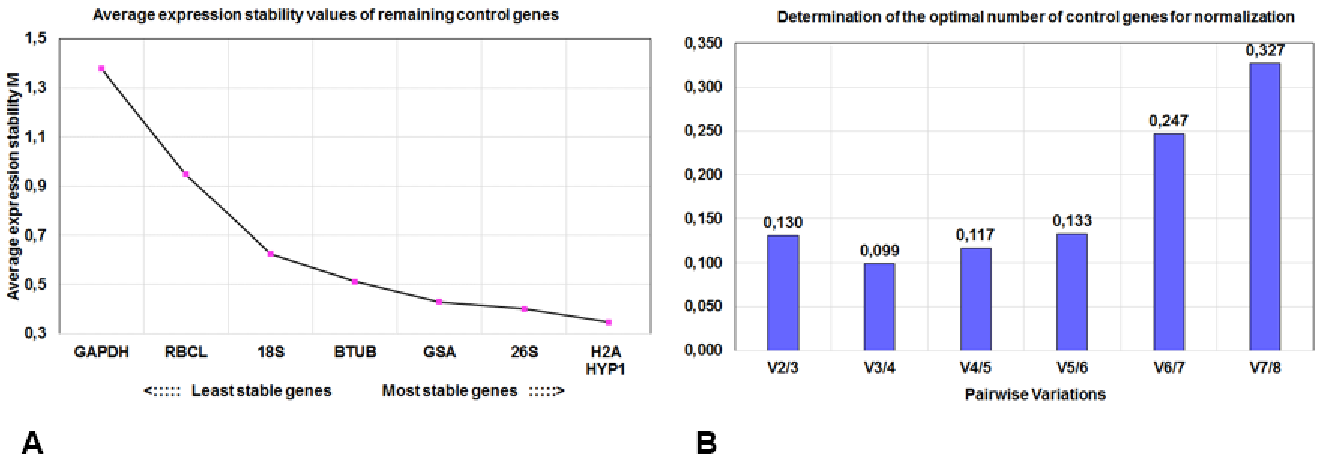

Figure S10. Expression stability analysis of candidate reference genes by GeNorm algorithm. (**A**) Average expression stability M. (**B**) Determination of the optimal number of reference genes for normalization by pairwise variation (*V*).

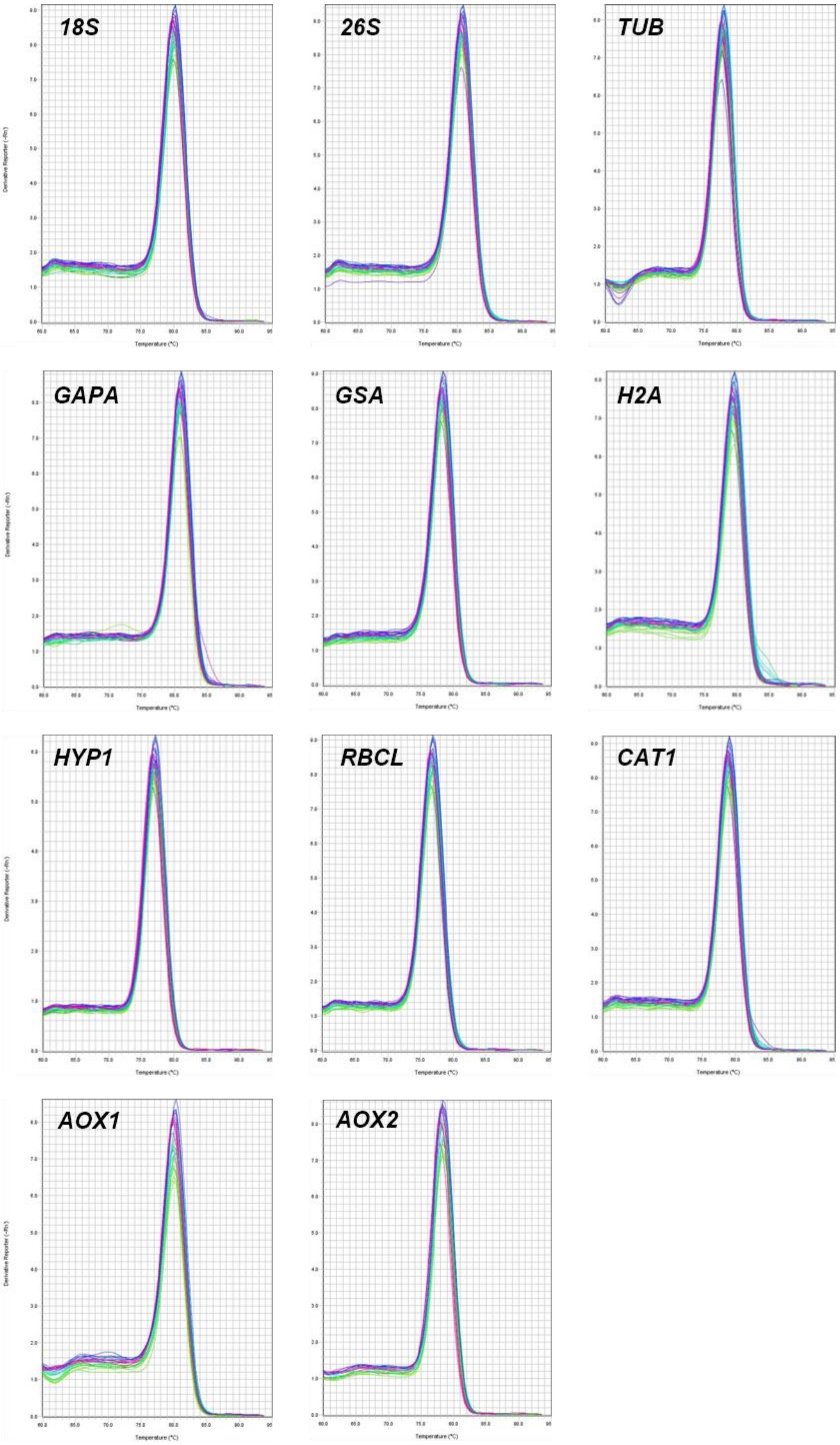
18S
26S
TUB
GAPA
GSA
H2A
HYP1
RBCL
CAT1
AOX1
AOX2

Figure S11. Melting curves of the genes analyzed showing specific peaks.

1.2 Supplementary Tables

Table S1. Primer sequences used in *HpAOX* genes isolation.

Gene	Aim	Primer sequence (5'-3')	Ta (°C)
HpAOX1	3'RACE-PCR	Fw: GCAGGGCGCCTTCTTCAACATCTAC	58
	5'RACE-PCR	Rev: CATCAAGTGCATCCGCTCGTTCTCT	68
	Whole gene	Fw: CCTAACTGCTCTCCTAAAAAGA<br>Rev: CTCTCTCCAACGAAGCAATG	55
HpAOX2	3'RACE-PCR	Fw: AAGCTCGCTCACAGGGTAACAGG	55
	5'RACE-PCR	Rev: GAACAGCCCCTGCACGATAAGAACC	68
	Whole gene	Fw: AGAAGGGGAATTTGTGGAG<br>Rev: CAACACAAGCTGAAGTAAGAAC	60

Ta: annealing temperature, Fw: forward, Rev: reverse.

Table S2. Eudicot plant species used in the NJ analysis.

O	F	Species	Accession number
Brassicales	Brassicaceae	*Arabidopsis lyrata*	AL1G33660<br>AL3G24680<br>AL3G24690<br>AL5G06730<br>AL0G08000<br>AL8G30860
		Arabidopsis thaliana	AT1G32350<br>AT3G22360<br>AT3G22370<br>AT3G27620<br>AT5G64210
		Boechera stricta	Bostr.19424s0421.1<br>Bostr.19424s0422.1<br>Bostr.3359s0124.1<br>Bostr.0568s0383.1
		Brassica rapa	Bra010153<br>Bra001865<br>Bra031351<br>Bra023835<br>Bra037768
		Capsella grandiflora	Cagra.1189s0011.1<br>Cagra.1189s0012.1<br>Cagra.5575s0007.1<br>Cagra.3957s0019.1<br>Cagra.0248s0102.1
		Capsella rubella	Carubv10014065m<br>Carubv10019557m<br>Carubv10009739m<br>Carubv10026681m
		Eutrema salsugineum	Thhalv10021013m<br>Thhalv10009342m<br>Thhalv10005697m
		Thellungiella parvula	TP3G20130<br>TP3G20140<br>TP2G27270
	C	*Carica papaya*	evm.model.supercontig_8.29<br>evm.model.supercontig_42.47
Curcubi	Curc	*Citrullus lanatus*	CL10G07420
		Cucumis melo	CM00136G00010
		Cucumis sativus	Cucsa.398150.1
Fabales	Fabaceae	*Glycine max*	GM04G14800<br>GM08G07690<br>GM08G07700<br>GM05G24455
		Lotus japonicus	LJ2G020780<br>LJ4G005280<br>LJ4G005290
		Medicago truncatula	MT5G026620<br>MT5G070680<br>MT5G070870<br>MT5G070880
		Phaseolus vulgaris	Phvul.002G127100.1<br>Phvul.002G209100.1<br>Phvul.002G209200.1

O	F	Species	Accession number
**Malvales**	**Malvaceae**	*Gossypium raimondii*	Gorai.008G296600.1<br>Gorai.012G142200.1<br>Gorai.005G220400.1<br>Gorai.005G220500.1
		Theobroma cacao	TC03G031300<br>TC02G011670
**Malpiahiales**	**Lina**	*Linum usitatissimum*	Lus10035670<br>Lus10005372<br>Lus10020523
	**Salicaceae**	*Populus trichocarpa*	PT03G09340<br>PT12G01430<br>PT12G01440<br>PT15G01960
		Salix purpurea	SapurV1A.1470s0080.1<br>SapurV1A.0346s0170.1<br>SapurV1A.0377s0140.1<br>SapurV1A.0377s0150.1<br>SapurV1A.3352s0030.1<br>SapurV1A.0894s0160.1
	**E**	*Manihot esculenta*	ME10292G00060
		Ricinus communis	RC30063G00030
**Ranunc**	**R**	*Aquilegia coerulea Goldsmith*	Aquca_105_00003.1<br>Aquca_043_00024.1<br>Aquca_008_00134.1<br>Aquca_033_00110.1
**Rosales**	**Rosaceae**	*Fragaria vesca*	FV5G29310<br>FV5G21950
		Malus domestica	MD00G028680<br>MD00G081720<br>MD13G026910<br>MD16G016620
		Prunus persica	Prupe.5G018700.1<br>Prupe.1G061800.1<br>Prupe.1G061900.1<br>Prupe.1G061400.1
**Sapindales**	**Rutaceae**	*Citrus clementina*	Ciclev10001766m<br>Ciclev10003687m<br>Ciclev10028835m
		Citrus sinensis	orange1.1g037339m<br>orange1.1g019765m<br>orange1.1g020532m
**Solanales**	**Solanaceae**	*Solanum lycopersicum*	Solyc08g005550<br>Solyc08g075540<br>Solyc08g075550<br>Solyc01g105220
		Solanum tuberosum	PGSC0003DMT400019708<br>PGSC0003DMT400019707<br>PGSC0003DMT400047562<br>PGSC0003DMG400012558
**A**	**a**	*Amborella trichopoda*	ATR_00038G01180<br>ATR_00048G01570
**B**	**b**	*Beta vulgaris*	BV5G19180<br>BV9G03180
**C**	**c**	*Kalanchoe laxiflora*	Kalax.0453s0008.1<br>Kalax.1476s0004.1<br>Kalax.0496s0017.1<br>Kalax.0907s0011.1<br>Kalax.0414s0014.1

D	***d***	*Mimulus guttatus*	Migut.J01127.1<br>Migut.E01358.1<br>Migut.N01067.1
E	***e***	*Eucalyptus grandis*	Eucgr.E01214.1<br>Eucgr.E01213.1<br>Eucgr.I02663.1
F	***f***	*Vitis vinifera*	VV02G09030<br>VV02G09050<br>VV00G00110

O: order, F: family; C: *Caricaceae*, Curcubi: *Curcubitales*, Curc: *Curcubitaceae*, Lina: *Linaceae*, E: *Euphorbiaceae*, Ranun: *Ranunculales*, R: *Ranunculaceae*, A: *Amborellales*, B: *Caryophyllale*, C: *Saxifragales*, D: Lamiales, E: Myrtales, F: *Vitales*, a: *Amborellaceae*, b: *Amarantaceae*, c: *Crassulaceae*, d: *Phrymaceae*, e: *Myrtaceae*, f: *Vitaceae*.

In grey the AOX2 sequences.

Table S3. Monocot plant species used in the NJ analysis.

Species	Accession number
Brachypodium distachyon	BD3G52505<br>BD5G20540<br>BD5G20547<br>BD5G20557
Hordeum vulgare	CAJW010038523<br>CAJW011587016<br>CAJW010099492
Musa acuminata	GSMUA_Achr5G03810_001<br>GSMUA_Achr6G01170_001<br>GSMUA_Achr6G01300_001<br>GSMUA_Achr1G27800_001
Oryza brachyantha	OB02G22630<br>OB02G36280<br>OB04G30980<br>OB04G30990
Oryza glaberrima	ORGLA02G0249500<br>ORGLA04G0206000<br>ORGLA04G0206100
Oryza sativa	BGIOSGA008063<br>BGIOSGA005788<br>BGIOSGA014421<br>BGIOSGA014422
Sorghum bicolor	SB04G030820<br>SB06G027410<br>SB06G027420<br>SB06G027430
Zea mays	ZM02G05480<br>ZM02G05490<br>ZM02G05500<br>ZM05G37570
